# Carrier-free multifunctional nanomedicine for intraperitoneal disseminated ovarian cancer therapy

**DOI:** 10.1186/s12951-022-01300-4

**Published:** 2022-02-22

**Authors:** Xiuyu Huang, Miaojuan Qiu, Tianqi Wang, Binbin Li, Shiqiang Zhang, Tianzhi Zhang, Peng Liu, Qiang Wang, Zhi Rong Qian, Chengming Zhu, Meiying Wu, Jing Zhao

**Affiliations:** 1 The Seventh Affiliated Hospital of Sun Yat-Sen University, Sun Yat-Sen University, Shenzhen, 518107 Guangdong People’s Republic of China; 2 School of Pharmaceutical Sciences (Shenzhen), Sun Yat-Sen University, Shenzhen, 518107 Guangdong People’s Republic of China

**Keywords:** Nanomedicine, Self-assembly, THZ1, Alendronate, Ovarian cancer

## Abstract

**Background:**

Ovarian cancer is the most lethal gynecological cancer which is characterized by extensive peritoneal implantation metastasis and malignant ascites. Despite advances in diagnosis and treatment in recent years, the five-year survival rate is only 25–30%. Therefore, developing multifunctional nanomedicine with abilities of promoting apoptosis and inhibiting migration on tumor cells would be a promising strategy to improve the antitumor effect.

**Methods and results:**

In this study, we developed a novel ACaT nanomedicine composed of alendronate, calcium ions and cyclin-dependent kinase 7 (CDK7) inhibitor THZ1. With the average size of 164 nm and zeta potential of 12.4 mV, the spherical ACaT nanoparticles were selectively internalized by tumor cells and effectively accumulated in the tumor site. Results of RNA-sequencing and in vitro experiments showed that ACaT promoted tumor cell apoptosis and inhibited tumor cell migration by arresting the cell cycle, increasing ROS and affecting calcium homeostasis. Weekly intraperitoneally administered of ACaT for 8 cycles significantly inhibited the growth of tumor and prolonged the survival of intraperitoneal xenograft mice.

**Conclusion:**

In summary, this study presents a new self-assembly nanomedicine with favorable tumor targeting, antitumor activity and good biocompatibility, providing a novel therapeutic strategy for advanced ovarian cancer.

**Graphical Abstract:**

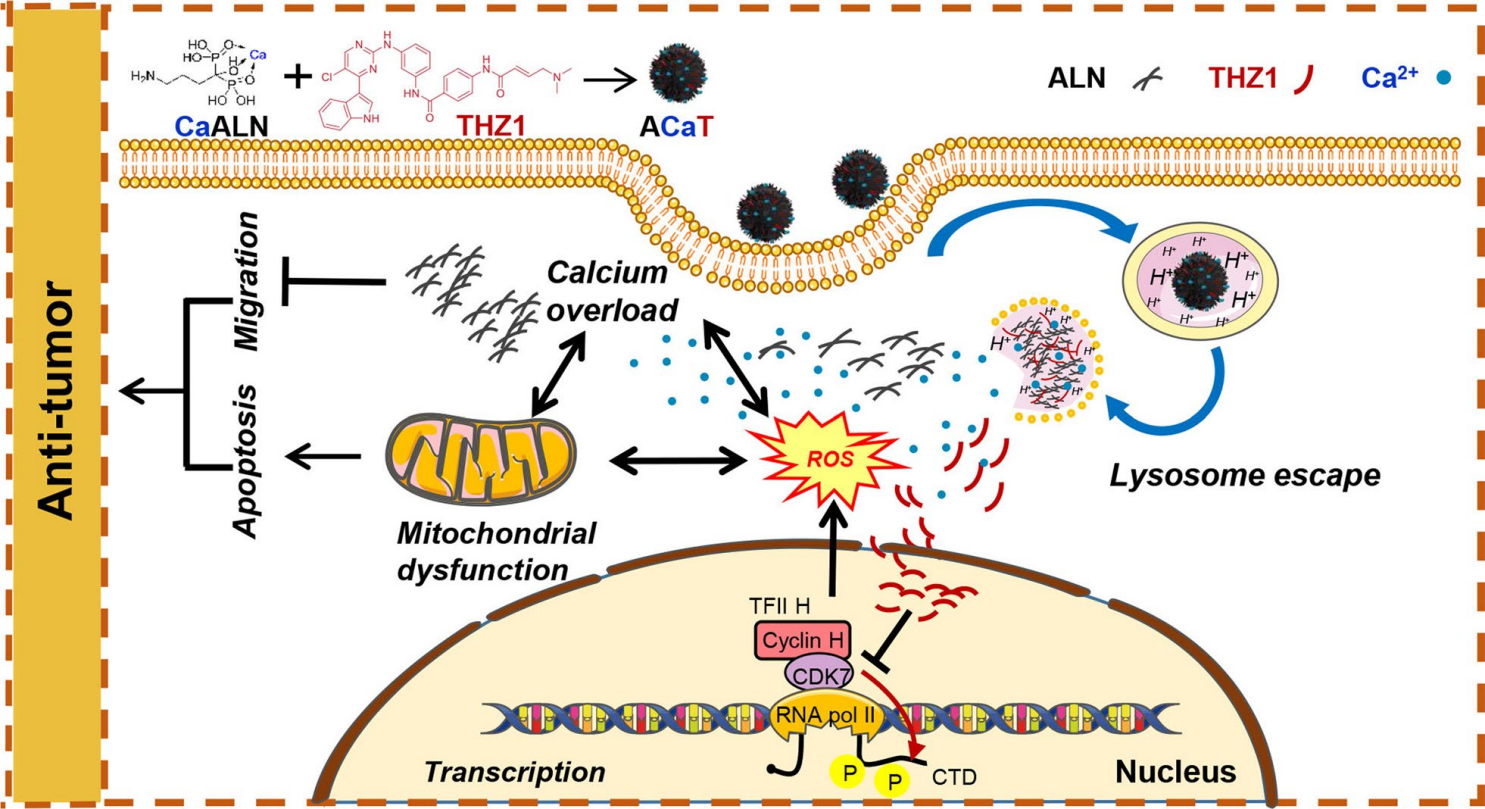

**Supplementary Information:**

The online version contains supplementary material available at 10.1186/s12951-022-01300-4.

## Background

Ovarian cancer (OC) is a common malignant tumor of female reproductive organs [1], which is often diagnosed in advanced stage [2], and easy to be widely implanted and transferred to the pelvic and abdominal cavity, forming malignant ascites [3]. Despite advances in diagnosis and treatment in recent years, the 5-year survival rate is only 25–30%, which is the lowest among all gynecological malignancies [4]. Most notably, malignant ascites of ovarian cancer is the result of peritoneal infiltration and metastasis of tumor cells, which seriously affects the quality of life of patients and is one of the main causes of death of patients [5]. The treatment of advanced ovarian cancer largely depends on surgical and platinum-based chemotherapy. Over the past decades, intraperitoneal chemotherapy in advanced ovarian cancer has the potential to improve cytotoxicity and inhibit ascites production by increasing tumor exposure to antineoplastic agents [6]. Platinum-based drugs, paclitaxel and mitomycin are commonly used in clinical and experimental intraperitoneal infusion [7, 8]. However, the small molecule drugs enter the circulatory system through the peritoneum-vascular barrier, resulting in systemic side effects and less tumor accumulation. In addition, drug resistance is the most common problem for recurrence of advanced ovarian cancer [9].

Recent studies have shown that the continuously active transcription of some oncogenes, such as MYC, is driven by large super-enhancers regions that are densely occupied by transcription factors and co-factors [10]. Thus, suppression of tumor cell proliferation by inhibiting super-enhancers attracts a huge attention of the clinical society. Cyclin-dependent kinase 7 (CDK7) is a component of transcription factor II H (TFIIH) and it mediates transcriptional dependence of important gene clusters associated with super-enhancers [11]. CDK7 inhibitor THZ1, a novel transcription-targeting compound, has been reported to significantly reduce the activity of super-enhancers and associated oncogene transcription factors through binding with CDK7 [12]. THZ1 has been demonstrated to have extensive cytotoxicity against ovarian cancer cell lines [13]. Notably, THZ1 showed excellent in vivo activity in patient-derived xenograft (PDX) mouse models of platinum and PARPi resistant ovarian cancer [10]. However, short half-life, poor bioavailability, and potential toxicity of THZ1 are drawbacks to be overcome. The half-life of THZ1 is only 45 min in mouse plasma, which limits its clinical application [14]. Therefore, developing a strategy that could improve bioavailability, prolong half-life time, and reduce potential toxicity, may increase the opportunity for the therapeutic application of THZ1.

Accompanying with the development of nanotechnology, nanomaterial has been used for cancer diagnosis and treatment in clinical trials [15–19]. An intraperitoneal chemotherapy study using xenograft mice demonstrated that the paclitaxel-loaded nanoparticles, compared to free paclitaxel, exhibited a 3.2-fold increase residence time in peritoneal cavity [20]. Injecting cisplatin-loaded nanoparticles showed enhanced antitumor activity in a rat of peritoneal carcinomatosis (PC), compared to free cisplatin [21]. The combination of nanostructured protein inhibitor and cisplatin by intraperitoneal injection could effectively downregulate tumor-promoting protein in metastatic ovarian cancer and ascites, and improve the survival rate of mice with metastatic ovarian cancer [22]. These results suggested that nanomedicine had stronger antitumor effects and a long-time accumulation in the abdominal cavity. Carrier-free nanomedicines have drawn great attention due to the high load rate and non-toxic side effects of exogenous carriers [23, 24]. In vivo studies have shown that nanomedicine exhibited longer blood half-life, better tumor selectivity, enhanced tumor accumulation, and significantly improved antitumor efficacy compared with free drugs [25, 26].

The current carrier-free nanomedicines mostly suppress tumor growth by inhibiting tumor cell proliferation, and few can inhibit the metastasis and invasion of tumor. Therefore, a multifunctional nanomedicine which can simultaneously inhibit tumor cell proliferation and migration is highly demanded. Alendronate, a nitrogen-containing bisphosphonate, is clinically used for the treatment of osteoporosis and bone metastasis [27]. It has been reported that alendronate could inhibit the mevalonic acid pathway by reducing Rho activation and has an effect on reducing tumor burden and ascites, thus inhibiting ovarian cancer cell migration [28].

Calcium ions are well known as an important second messengers that regulate many cellular functions. Intracellular calcium levels are balanced by various physiological metabolic processes [29]. Changes in intracellular calcium concentration caused by non-cellular physiological regulation can interfere with calcium homeostasis and affect cell activity [30]. Compared with normal cells, cancer cells with abnormal metabolism usually produce a high level of ROS, which regulates cellular calcium signal transduction, extending the opening time of mitochondrial permeability transition pores, and leading to a rapid increase in mitochondrial intima permeability. Mitochondrial swelling causes rupture of the outer membrane, inducing the release of intermembrane proteins and initiating pathways of cell death such as apoptosis and necrosis [31–33]. ROS inducer THZ1 may act synergistically with Ca^2+^. On the one hand, it can cause calcium overload by introducing excessive Ca^2+^; on the other hand, THZ1 promotes intracellular ROS production, synergistically destroys calcium homeostasis and triggers apoptosis of cancer cells [34].

Herein, an ACaT carrier-free nanomedicine, which was composed of alendronate, THZ1 and Ca^2+^, was designed for intraperitoneal disseminated ovarian cancer therapy. Alendronate and Ca^2+^ formed network structure through coordination interaction, and THZ1 was self-assembled into the structure by hydrophobic attraction. This carrier-free nanomedicine could specifically target tumor and simultaneously achieve anti-migration and pro-apoptosis in an intraperitoneal xenograft model with human ovarian cancer. Moreover, ACaT could be degraded in low pH and showed good biocompatibility. Overall, this novel carrier-free nanomedicine ACaT provides new enlightenment for the clinical treatment of ovarian cancer.

## Materials and methods

### Materials

Calcium chloride dihydrate (CaCl_2_·2H_2_O), alendronate sodium trihydrate (NaALN·3H_2_O) were purchased from Shanghai Aladdin Bio-Chem Technology Co., LTD (Shanghai, China). CDK7 inhibitor THZ1 and the cell counting kit-8 (CCK8) assay kit were purchased from MedChemExpress (MCE, Shanghai). Dimethyl sulfoxide (DMSO) and penicillin–streptomycin (pen/strep) solution (100 ×) were purchased from Sigma Aldrich (MO, USA). Cell culture medium, trypsin–EDTA and fetal bovine serum (FBS) were provided by Gibco (Guangzhou, China). Annexin V-FITC/PI apoptosis detection kits and mitochondrial membrane potential detection kit were purchased from Beyotime (Shanghai, China). 6-carboxy fluorescein (6-FAM), DAPI dihydrochloride and DiR iodide were purchased from Invitrogen (Carlsbad, CA, USA). D-Luciferin potassium salt and Fluo-4, AM were acquired from Yeasen Biotech Co., Ltd (Shanghai, China). Acridine orange-ethidium bromide (AO/EB) staining kit was purchased from Leagene Biotechnology (Beijing, China). Bcl-2, Bax and cleaved caspase-3 antibodies were bought from Absin Bioscience (Shanghai, China). β-actin and secondary antibodies were purchased from Proteintech (Wuhan, China). BCA protein quantitation kit and enhanced chemiluminescence (ECL) kit were purchased from Perkin-Elmer (Waltham, USA). All other reagents were consistent with previous report [35].

### Cell culture and animal

SKOV3 cells (human ovarian cancer cell line), human renal proximal convoluted tubule epithelial cells HK2 and human peritoneal mesenchymal cells HMrSVS were purchased from American Type Culture Collection (ATCC, USA) in May 2016. All cells were routinely tested for mycoplasmas contamination using a mycoplasma detection kit (Beyotime, China) before used. SKOV3, HK2 and HMrSVS cultured in McCoy’s 5A, DMEM/F12 and RPMI 1640 medium with 10% fetal bovine serum (FBS) and 1% pen/strep solution in a humidified atmosphere of 5% CO_2_ and 95% air at 37 °C. Firefly luciferase labeled SKOV3 cells (SKOV3-Luc) was generated as previously described [18]. Five-week-old to six-week-old female BALB/c nude mice (18–22 g) were purchased from GemPharmatech Co., Ltd (Guangzhou, China). All animal experiments were approved by the Animal Care and Use Committee (SYSU-IACUC-2021-B0815) of Sun Yat-sen University.

### Preparation and characterization of ACaT

ACaT were prepared through the self-assembly of THZ1, NaALN and Ca^2+^. Firstly, 0.66 g of NaALN·3H_2_O and 0.2 g of CaCl_2_·2H_2_O were dissolved in 75 mL of water, respectively, and mixed them up. Then, 50 mg of THZ1 (4 mg/mL) was added to the above solution. The pH of this mixture was adjusted to 7.0 by NaOH and stirred for 30 min at 4 °C. ACaT nanomedicine was obtained by washing and centrifugation. The morphologies of obtained formulations were observed by TEM and SEM. Particle size and zeta potential were measured by Zetasizer Nano ZS90 (Malvern, Britain). Fourier transform infrared (FTIR) spectra were measured using Thermo Scientific Nicolet iS5 FTIR spectrometer. Chemical state and composition of ACaT was investigated using X-ray photoelectron spectrometer (Thermo Scientific, K-Alpha, UK). To assess the stability of ACaT in PBS, PBS containing 5% and 10% FBS, the particle sizes were measured by DLS persistently at different time. X-ray powder diffraction (XRD) patterns were recorded using an X-ray diffractometer (Rigaku D/max 2550 V, Japan). UV–vis and fluorescence spectra were recorded by Evolution 300 UV–vis (Thermo Scientific, USA) and LS55 luminescence spectrometer (Perkin-Elmer, USA).

### Degradation and in vitro drug release test

The degradation of ACaT was calculated by monitoring the Ca centration. 10 mg ACaT was dispersed in 10 mL PBS and the Ca centration was detected by an ion monitor (S220B Seven Compact™, Mettler Toledo, Switzerland). For THZ1 drug release, 10 mg ACaT was dispersed in 10 mL PBS (pH = 4.5, 6.0, 7.0) at 37 °C. Then, 100 μL of release media was extracted at different time points. Supernatant was removed after centrifugation. The amount of drug release from THZ1 was measured by High Performance Liquid Chromatography (HPLC). The HPLC was performed in C18 column, following a different gradient elution program with a flow rate of 1.0 mL/min. Mobile eluent A was HPLC-grade water and mobile eluent B was pure methanol (HPLC-grade). Before spectra acquisition, mobile eluent A, B and samples were passed through a 0.22 μm membrane filter to remove any particles and then sonicated for 10 min to remove air bubbles.

### In vitro cellular uptake of ACaT

ACaT nanoparticles were labeled with 6-FAM for 24 h at room temperature. In brief, SKOV3 cells were seeded in confocal dish and cultured until 60% density. Cells were then incubated with ACaT/6-FAM at 37 °C for 1, 2, 4 and 24 h, respectively. After incubation, cells were washed twice with PBS and fixed with 4% paraformaldehyde for 8 min, then stained with 5 μg/mL of DAPI, and photographed by confocal laser scanning microscopy (CLSM, Zeiss LSM880, Germany).

### In vitro cytotoxicity study

The in vitro cytotoxicity of THZ1, NaALN and ACaT were tested by CCK8 cell proliferation assay kit. According to the manufacturer’s instructions, 5000 SKOV3 cells were seeded into a 96-well plate per well and cultured overnight. Then cells were incubated with various concentrations of THZ1, NaALN and ACaT for 24, 48 and 72 h, respectively. At each time point, 10 μL of CCK-8 reagent was added into each well and incubated for another 3 h. Optical density (OD) of 450 nm was measured by a microplate reader (BioTek, SynergyH1, USA). Cell viabilities were calculated using the formula: cell viability % = (OD_sample_ – OD_blank_/OD_control_ – OD_blank_) 100%.

### Analysis of cell apoptosis

Cell apoptosis was assessed via AO/EB staining, flow cytometry and western blot analysis. Briefly, SKOV3 cells were pre-seeded in 6 well plates (5 × 10^4^ cells per well) overnight. Then ACaT with varied concentrations were added into each well and incubated for 24, 48 or 72 h, respectively. The cells were stained by AO/EB assay kit at different time points. Then image acquisition and analysis were carried out by a fluorescent microscopy (Leica, DMi8, Germany). Apoptosis was also investigated by flow cytometry using annexin V/PI co-staining as described previously [35]. For western blot analysis, the SKOV3 cells were washed and lysed in 150 μL of lysis buffer containing phenylmethylsulfonyl fluoride (PMSF, 1 mM) to collect total proteins. Total protein concentrations were measured using BCA protein quantitation kit (Waltham, USA). The protein samples were separated by 10% sodium dodecyl sulfate polyacrylamide gel electrophoresis (SDS-PAGE) at 100 V for 30 min followed by 120 V for 60 min, and then electrotransferred to polyvinylidene fluoride (PVDF) membranes. Membranes were blocked with 5% defatted milk for 2 h and incubated with primary antibody overnight at 4 °C, followed by incubation with secondary antibody for 2 h at room temperature. After that, the membranes were visualized in ECL reagent for 1–5 min and western blot images were collected on ChemiDoc Imaging System (Bio-Rad, America).

### Analysis of migration

Briefly, 5 ×10^5^ cells were seeded per well overnight in 6-well culture plates until 80% confluency was achieved. On the second day, a vertical scratch was performed using a 10 μL pipette tip to draw horizontal four lines (0.5 cm apart) and one vertical line evenly with ruler, and the cells were washed twice using fresh PBS to remove the dead or floating cells. Subsequently, the cells were incubated with various concentrations of ACaT (6.25 mg/L), NaALN (200 μM), THZ1 (0.01 μM). The control group was incubated with culture medium. Scratches were imaged by Nikon camera at 0 and 24 h. Data were analyzed using *Image J* software.

### Measurement of intracellular calcium ion

Intracellular calcium ion accumulation was detected by using CLSM and Fluo-4 AM dye as fluorescent probe. SKOV3 cells were incubated with media containing ACaT, NaALN, and THZ1 for 48 h at concentration of 1.56 mg/L, 200 μM and 0.01 μM, respectively. The control group was incubated with media containing the same volume of PBS. After 48 h of treatment, media was removed and cells were stained with 1 μM of Fluo-4 AM for 60 min. The cells were washed with HBSS for 3 times, and then incubated in a 37 °C incubator for 30 min to ensure that Fluo-4 AM were completely transformed into Fluo-4 in the cells. And the fluorescence was recorded by CLSM at an excitation of 488 nm laser.

### Detection of mitochondrial membrane potential

The mitochondrial membrane potential was detected by a JC-1 based mitochondrial membrane potential kit. Cell culture in the same manner as described for migration assay above. The original media was removed, and 1 mL of JC-1 working solution was added. The cells were maintained at 37 °C/5% CO_2_ in an incubator for 20 min, washed twice with JC-1 washing buffer. Then 2 mL of cell medium was added. The samples were observed and photographed under a Leica DMI8 fluorescent microscope (Leica).

### mRNA extraction and sequencing

Cells were treated with THZ1 (0.5 μM), NaALN (850 μM), and ACaT (100 mg/L) for 24 h. After treatments, cells were washed with ice-cold PBS three times and total RNA was extracted using Qiagen RNeasy Mini kit (Germany) according to the manufacturer protocol. RNA samples were quantified using the Qubit 2.0 (Thermo Fisher Scientific) and treated with DNase I to remove residual DNA. The quality control of mRNA samples was conducted using the Bioanalyzer 2100 (Agilent Technologies). RNA-seq libraries were generated and sequenced following the standard mRNA protocols on a Hiseq 2500 (Illumina, PE150). Clean reads were obtained from the raw reads by removing the adaptor sequences, low quality sequences and reads containing poly-N with Trimmomatic software. All the downstream analyses were based on the clean data with high quality. DESeq2 was selected to identify differentially expressed genes (DEGs). Genes with an adjusted P value < 0.05 and abs (log_2_ (fold change)) > 1 were assigned as DEGs. Gene set enrichment analysis (GSEA) was performed using the Kyoto Encyclopedia of Genes and Genomes (KEGG) pathways.

### ACaT biodistributions in vivo

SKOV3-Luc cells were trypsinized, washed, resuspended with ice-cold PBS, and kept on ice prior to implantation in mice. Peritoneal tumor model was initiated in mice by intraperitoneal injecting 5 × 10^6^ SKOV3 cells per mouse intraperitoneal. ACaT nanomedicine was prelabeled with DiR dye. To further evaluate the biodistribution of ACaT, SKOV3 tumor-bearing BALB/c nude mice were intraperitoneally injected with free DiR and ACaT-DiR, and then monitored by real time in vivo imaging system (IVIS). Mice were imaged with an IVIS at 1, 4, 24, 72 and 168 h post injection. At 72 h and 168 h post injection, mice were sacrificed, and tumors as well as major organs were collected and fluorescently imaged immediately. The quantitative analyses of fluorescence signals were performed using *AnitView100*.

### Antitumor effect of ACaT in vivo

When peritoneal metastasis model was established after 5 days, the tumor-bearing mice were randomly divided into four groups (*n* = 8) and intraperitoneally administered with PBS, NaALN (8 mg/kg), THZ1 (1 mg/kg) and ACaT (10 mg/kg) in 400 uL of PBS solution for 8 cycles. The body weights and abdomen circumference in mice were measured once a week. An *AnitView100* IVIS imaging system was used for live mice imaging at one week interval. Five mice were fixed and subjected to weekly bioluminescent imaging. The other three mice in each group were killed under deep anesthetized with an overdose of sodium pentobarbital and dissected on day 35. Ascites volumes were measured and the excised tumors were photographed and weighed. Tumors and organs such as heart, liver, spleen, lung and kidney were collected for H&E and TUNEL staining.

### Biocompatibility assessment in vitro and in vivo

HK2 and HMrSVS cells were incubated on 96-well plates overnight and ACaT with different concentrations were added and incubated at 37 °C for 24, 48 and 72 h, respectively. In addition, hemolytic activity test was also performed at the same time. The fresh red cells were constituted with normal saline (0.9%) into 2% red blood cell suspension, and mixed with different concentrations of ACaT solutions at 37 °C for 4 h. The negative control was incubated with normal saline (0.9%), while the positive control was incubated with sterilized water. After incubation, all groups were centrifuged at 1500 rpm for 5 min. For each concentration, 100 μL of supernatant was added to a 96-well plate and absorbance at 570 nm were detected by microplate reader. HR% = (A_sample_ – A_negtive_) / (A_positive_ – A_negtive_) 100% [35]. To evaluate the biocompatibility of ACaT in vivo, BALB/c mice were sacrificed at 72 h after injected with ACaT nanomedicine. Organs such as heart, liver, spleen, lung and kidney were collected for H&E staining.

### Statistical analysis

Data are shown as the average (± SD) taken from at least three independent experiments. Statistical analysis was performed using Prism 8.0 (GraphPad). Statistical differences between the values were evaluated using one-way ANOVA analysis of variance.

## Results and discussion

### Characterizations of ACaT

**Fig. 1:**
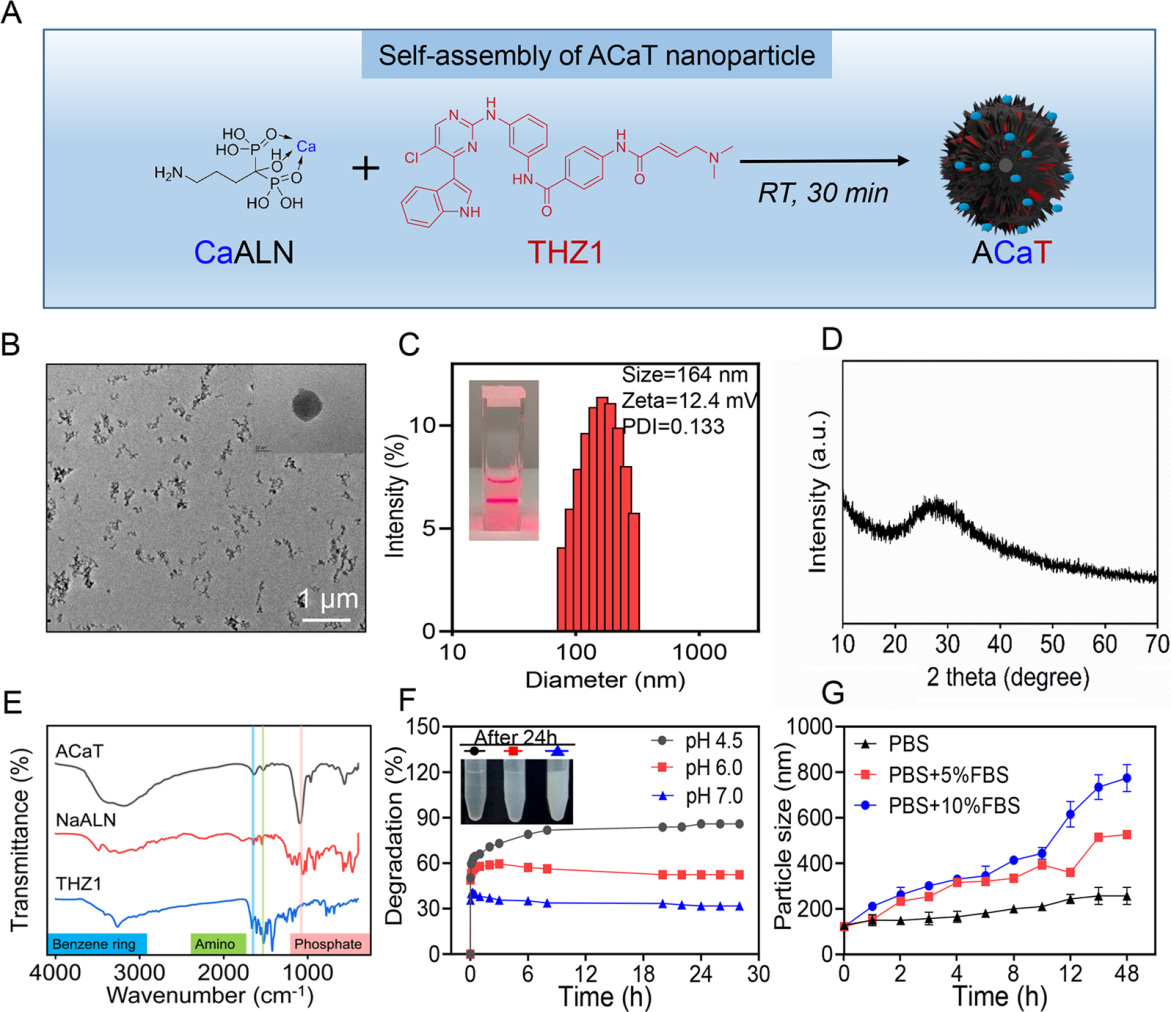
Characterizations of ACaT nanomedicine. **A** Schematic representation, **B** TEM images, **C** size distribution, **D** XRD pattern, and **E** FTIR spectra of alendronate sodium, THZ1 and ACaT. **F** Degradation of ACaT nanoparticles in PBS at different pH values. **G** Stability of ACaT nanomedicine in different media

Alendronate-calcium-THZ1 (ACaT) nanomedicine was prepared by the self-assembly of alendronate, Ca^2+^, and THZ1. As shown in Fig. 1A, alendronate and Ca^2+^ formed infinite coordination structure through coordination interaction, and THZ1 were self-assembled into the structure by hydrophobic attraction. The obtained ACaT exhibited amorphous structure and spherical morphology with average particle size of 164 nm (Fig. 1B–D). In the process of synthesis, the phosphate groups of alendronate sodium and Ca^2+^ can form CaALN clusters in the solution. The growth of CaALN clusters was controlled by regulating the ratio of alendronate to Ca^2+^ and the reaction time, temperature and pH. Zeta potential of ACaT was 12.4 mV, which could be easily internalized by cancer cells. Fourier transform Infrared (FTIR) analysis was employed to verify the existence of alendronate and THZ1. As shown in Fig. 1E, the FTIR spectra presented the characteristic peaks of phosphate group located at 1000–1200 cm^−1^ (the red stripe), which related to alendronate (ALN) were fit well with that of ACaT [36, 37]. The characteristic peaks of amino group (1520 cm^−1^, the green stripe) [38] and benzene ring (1620 cm^−1^, the blue stripe) [39] indicated the formation of the THZ1 structure.

To obtain further insight, the X-ray photoelectron spectroscopy (XPS) was used to analyze the chemical state and composition of the elements. As shown in Additional file 1: Fig. S1, the XPS survey spectra of ACaT samples showed Ca2p, C1s, N1s and P2p signals at approximately 347, 285, 401 and 133 eV, respectively, indicating the presence of Ca, N, O, P elements in the ACaT samples. The degradation of nanoparticles was detected by incubating ACaT with PBS at different pH values, indicating that the degradation rate of ACaT in acidic (pH = 4.5, 6.0) environment was faster than that in neutral (pH = 7.0) condition (Fig. 1F). It was suggested that in the acidic lysosomal environment (pH = 4.5), ACaT could be almost completely degraded, accompanied by the release of alendronate and THZ1 to cytoplasm. To further confirm the THZ1 releases from ACaT, THZ1 concentrations at different time points in ACaT supernatants were quantified by HPLC. As shown in Additional file 1: Fig. S2, 31.48 ± 4.85% of the THZ1 was released from the ACaT after 24 h at pH 7.0, while cumulative release amount exceeded 50.15% and 69.87% at pH 6.0 and pH 4.5. THZ1 release was significantly accelerated at 12–24 h and reached the maximum at 48 h.

In physiological environment (pH = 7.0 ~ 7.4), the degradation of nanoparticles was slow, and the size of nanoparticles remained relatively stable for over 72 h in PBS with pH of 7.0. However, in FBS-containing PBS, we found that the particle size of ACaT increased over time. Particle size of 10% FBS increases faster than 5% FBS. At 5% and 10% FBS-containing PBS with pH of 7.4 for 72 h, the average particle size finally stabilized at about 650 nm (Fig. 1G). The main reason was that the adsorption of serum protein onto nanoparticles leads to change of surface properties and the increasement of particle size. Collectively, a novel pH-sensitive carrier-free nanomedicine ACaT with mean size of 164 nm and positive surface charge was successfully prepared.

### Therapeutic effects of THZ1 and alendronate in vitro

**Fig. 2:**
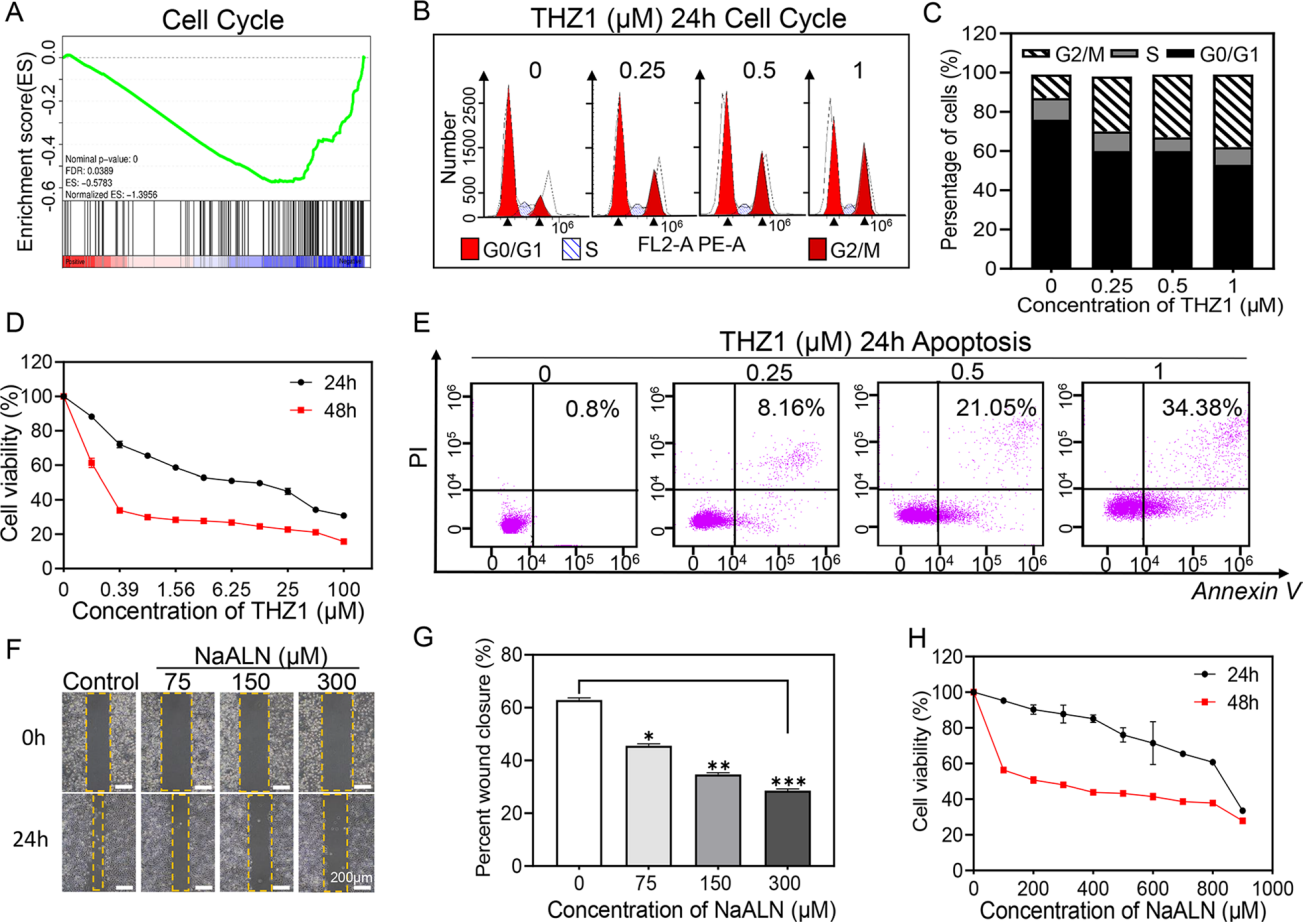
Therapeutic effect and mechanism of THZ1 and Alendronate in vitro. **A** GESA plot for genes of cell cycle after THZ1 treatment. **B** Flow cytometric analysis of SKOV3 cells treated with THZ1 for 24 h at different concentrations. **C** Quantitative cell cycle analysis of different phases in THZ1 treatment groups with different concentrations. **D** Cell viability of SKOV3 treated with THZ1 at different concentrations for 24 and 48 h. **E** Detection of apoptotic SKOV3 cells after treatment with THZ1 at concertrantion of 0, 0.25, 0.5 and 1 μM for 24 h by flow cytometry. **F** Scratch assay for cell migration. Scratch edges were recorded at 24 h after scratching and treating with alendronate at concentration of 75, 150 and 300 μM (yellow dotted boxes marked the scratch edges). **G** Quantification of the percentage of the wound area in scratch migration assay. **H** Cell viability of SKOV3 treated with different concentrations of NaALN for 24 and 48 h

THZ1 have been reported to downregulate the expression of super-enhancer associated genes by inhibiting CDK7, which in turn block cell cycle and transcription of cancer cells [40]. Transcriptome sequencing was performed on THZ1 treated SKOV3 cells (Additional file 1: Fig. S3). The gene set enrichment analysis (GSEA) showed that genes related to cell cycle were concentrated, and differential genes were enriched in down-regulated regions (Fig. 2A), indicating that THZ1 might perturb cell cycle progression. Flow cytometry was used to evaluate cell cycle distribution after treatment with different concentrations of THZ1. The results showed that the proportion of SKOV3 cells in G2/M phase was elevated with increased THZ1 concentration, suggesting the G2/M phase cell-cycle was arrested, which was consistent with the results of previous studies (Fig. 2B, C) [41, 42]. Disruption of cancer cell cycle is known to inhibit cell growth and induce apoptosis. As shown in Fig. 2D, E, THZ1 could significantly inhibit cancer cell proliferation and induce apoptosis at low concentration (1 μM).

Alendronate, an FDA-approved drug for the treatment of osteoporosis, has been reported to inhibit tumor metastasis [43]. Figure 2F, G showed that cancer cell migration ability was significantly suppressed after treatment with different concentrations of alendronate. The KEGG Orthology Based Annotation System (KOBAS) database was utilized to identify KEGG pathway enrichment of DEGs. Notably, focal adhesion pathway, which was associated with migration, was significantly downregulated (Additional file 1: Fig. S4). This observation was further confirmed in KEGG pathway map 04,510 (Additional file 1: Fig. S5). In addition, treatment with alendronate for 48 h also showed obvious growth inhibition effect on SKOV3 cells (Fig. 2H). The exact mechanism of apoptosis induced by alendronate still remains elusive. The previously reported alendronate treatment induced apoptosis in osteosarcoma cell through inhibiting PI3K-Akt-NFkappaB cell survival pathway [44].

### Antitumor effect and mechanism of ACaT in vitro

**Fig. 3:**
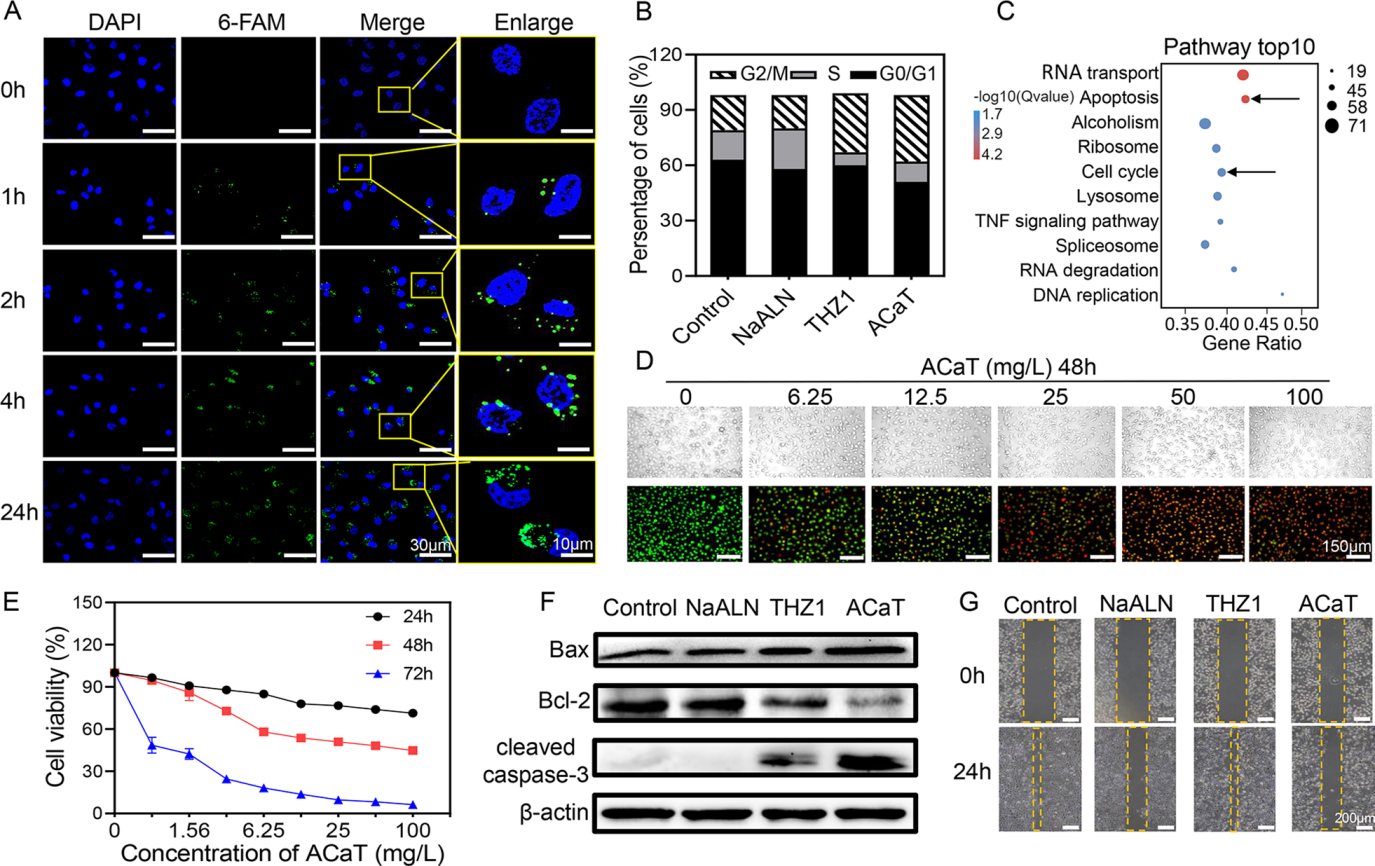
Evaluation of cellular uptake, antitumor activity and mechanism of ACaT in vitro. **A** CLSM images of SKOV3 cells after treatment with ACaT/6-FAM for 0, 1, 2, 4 and 24 h. Scale bar, 30 μm for the three columns on the left and 10 μm for the enlarge column. **B** Quantitative cell cycle analysis of different phases in different treatment groups. **C** Top 10 KEGG pathways enrichment analysis bubble-plot. Cells were treated with 100 mg/L of ACaT for 24 h before mRNA sequencing. Black arrow indicated cell apoptosis and cell cycle pathway. **D** Dual AO/EB fluorescent staining of SKOV3 cells after treatment with different concentrations of ACaT for 48 h (green for living cells and red for dead cells). Scale bar, 150 μm. **E** Cell viability of SKOV3 treated with ACaT at different concentrations for 24, 48 and 72 h. **F** Western blots for Bax, Bcl-2, and cleaved caspase-3 after NaALN (850 μM), THZ1 (0.5 μM), ACaT (100 mg/L) treatment for 24 h in SKOV3 cells. **G** Scratch assay for cell migration. Cell migration was recorded at 0 and 24 h after scratching. Cells were treated with NaALN (200 μM), THZ1 (0.01 μM), ACaT (6.25 mg/L) at the beginning of the experiment (yellow dotted boxes marked the scratch edges)

Antitumor drugs based on nanotechnology have been widely reported to change the pharmacokinetic characteristics and reduce system toxicity of small molecule drugs. In this study, ACaT nanomedicine was precisely designed for intraperitoneal disseminated cancer treatment. To investigate the uptake efficiency of ACaT by SKOV3 cells, ACaT was labelled with fluorescent probe 6-FAM (ACaT/6-FAM, green) to track the internalization in SKOV3 cells. The results showed that the intracellular uptake of ACaT by SKOV3 cells was time-dependent and the ACaT fluorescence intensity in the cytoplasm of SKOV3 was strong when the incubation time at 24 h (Fig. 3A), indicating that ACaT nanomedicine could be quickly and efficiently internalized, which was conducive to further evaluation of anticancer effect in vitro and in vivo.

In order to explore the antitumor effect and mechanism of ACaT in vitro, the cell proliferation and migration abilities were evaluated. Firstly, RNA transcriptome sequencing and flow cytometry were applied for assessing the cell cycle distribution. As shown in Fig. 3B and Additional file 1: Fig. S6, THZ1 and ACaT significantly inhibited the cell cycle process, making it arrested in G2/M phase. In addition to cell cycle, KEGG enrichment assay showed that cell apoptosis pathway in ACaT group was significantly affected as well. And cell apoptosis and cell cycle-related pathways were significantly enriched in bubble diagram (Fig. 3C), indicating that the THZ1 component in ACaT had significant effects on apoptosis and cell cycle. In addition, cell apoptosis was detected by AO/EB co-staining. As shown in Fig. 3D, E, ACaT exhibited time- and concentration-dependent killing against SKOV3 cells. The higher the ACaT concentration leads to the better killing effect. It has been reported that THZ1 treatment can increase intracellular reactive oxygen species levels due to reduced expression of NRF2 and glutathione biosynthesis genes, leading to apoptosis induction and cell death [45]. In addition to blocking cell cycle and inducing apoptosis, THZ1 was also demonstrated to induce intracellular ROS elevation (Additional file 1: Fig. S7). The increased ROS may lead to the disruption of mitochondrial membrane system function, calcium homeostasis disequilibrium, and eventually cell apoptosis [46]. In our study, calcium ion accumulation and mitochondrial membrane potential loss were observed in the ACaT group (Additional file 1: Figs. S8, S9). Calcium alone has little direct killing effect on SKOV3 cells (Additional file 1: Fig. S10). Mitochondrial dysfunction induced by Ca^2+^ overload may enhance the therapeutic effect of nanomedicines in cancer [47]. Therefore, we inferred that the increased ROS production and calcium accumulation facilitated each other in cell apoptosis.

Overall, ACaT induced cancer cell apoptosis through variety of mechanisms. Western blot was used to confirm the apoptosis of SKOV3 cells treated with different drugs for 24 h. As shown in Fig. 3F, the cells treated with both THZ1 and ACaT exhibited the increased pro-apoptotic protein Bax and the downregulated anti-apoptotic protein Bcl-2 to induce apoptosis. However, alendronate treatment had no effect on the expressions of Bax and Bcl-2, illustrating that alendronate contributed little to cell apoptosis. Cleaved-caspase-3, classical apoptosis marker, was detected in SKOV3 cell lysates after treatment with THZ1 and ACaT. We reasoned therefore that THZ1 was the primary effector of ACaT-induced apoptosis in SKOV3 cells. On the other hand, inhibiting migration of cancer cells by ACaT was verified by cell scratch assay (Fig. 3G and Additional file 1: Fig. S11). The results showed that both NaALN and ACaT significantly reduced the migration rate of SKOV3 cells. Generally, each component of ACaT retained its biological activity and exerted anticancer effects via multiple mechanisms.

### Distribution of ACaT in vivo

**Fig. 4:**
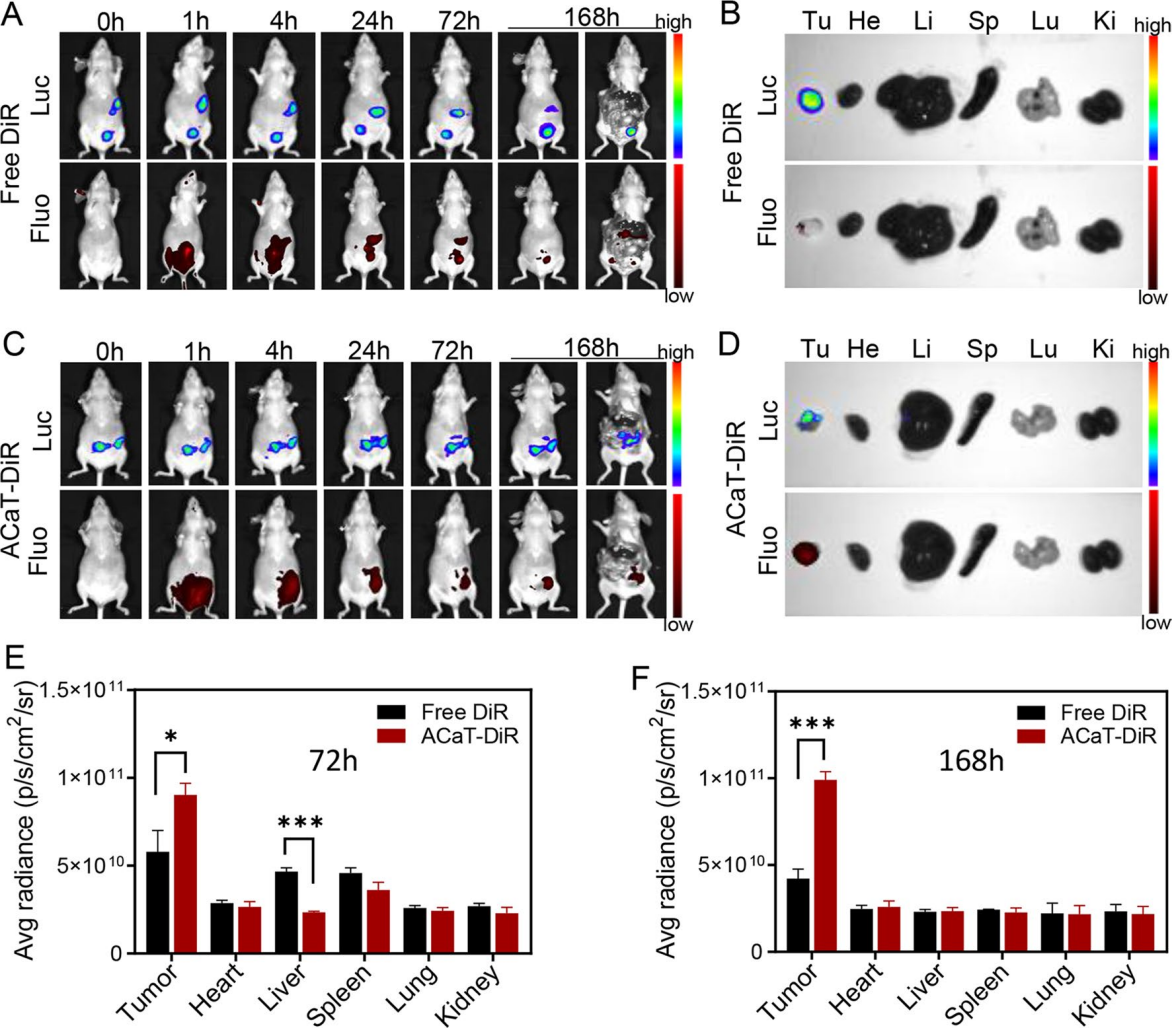
The in vivo biodistribution of free DiR and ACaT-DiR in female SKOV3-Luc tumor-bearing mice. The in vivo bioluminescent images (Luc) and fluorescent images (Fluo) of mice after intraperitoneal injection of (**A**) Free DiR and (**C**) ACaT-DiR at the indicated time points. The ex vivo bioluminescent images (Luc) and fluorescent images (Fluo) for tumor tissues and major organs of (**B**) free DiR and (**D**) ACaT-DiR at 168 h intraperitoneal injection. The quantitative analyses of pixel fluorescence intensity from tumor tissues and major organs after intraperitoneal injection 72 h (**E**) and168 h (**F**). *p < 0.05 and ***p < 0.005

In order to explore the biodistribution and anticancer effect of ACaT in ovarian cancer bearing mouse, SKOV3-Luc cells expressing firefly luciferase were used to establish an ovarian cancer peritoneal tumor model in BALB/c nude mice. The tumor progression could be conveniently detected by the in vivo imaging system. After 5 days of tumor cell transplantation, the mice were sacrificed, dissected and photographed. A solid tumor with a diameter of about 4 mm could be found in the lower right corner of the liver (Additional file 1: Fig. S12). Liver and spleen metastases began to appear around day 30 (Additional file 1: Fig. S13). The biological distribution of ACaT was studied on the 15 days after tumor transplantation. Free DiR and DiR labeled ACaT were intraperitoneally injected into mouse respectively. As shown in Fig. 4A, free DiR was distributed in the abdominal cavity at 1 h after injection, enriched in liver and tumor 72 h after injection (Fig. 4E and Additional file 1: Fig. S14). No obvious fluorescent signal was detected at 168 h, implying most of free DiR excreted out of the body 168 h after injection (Fig. 4B, F). ACaT-DiR was distributed throughout the abdominal cavity 1 h after injection (Fig. 4C), followed by preferentially accumulated in the tumor site even at 168 h post-injection (Fig. 4D–F).

Compared with small molecule compounds, the tumor target advantage of ACaT could be attributed to the existence of the peritoneal-plasma barrier, which maintained a high regional concentration of ACaT nanoparticles in the peritoneal cavity. Afterwards, designed ACaT nanoparticles with nanosize and positive-charge preferred to gather in tumor tissues due to the direct contact with tumor cells. By contrast, other intraperitoneal organs, such as liver, spleen, kidneys and intestines, are covered with peritoneum [48, 49], which is consist of closely arranged mesothelial cells [50] and not easily penetrated by nanoparticles.

### Antitumor effect of ACaT on human ovarian cancer bearing mice

**Fig. 5:**
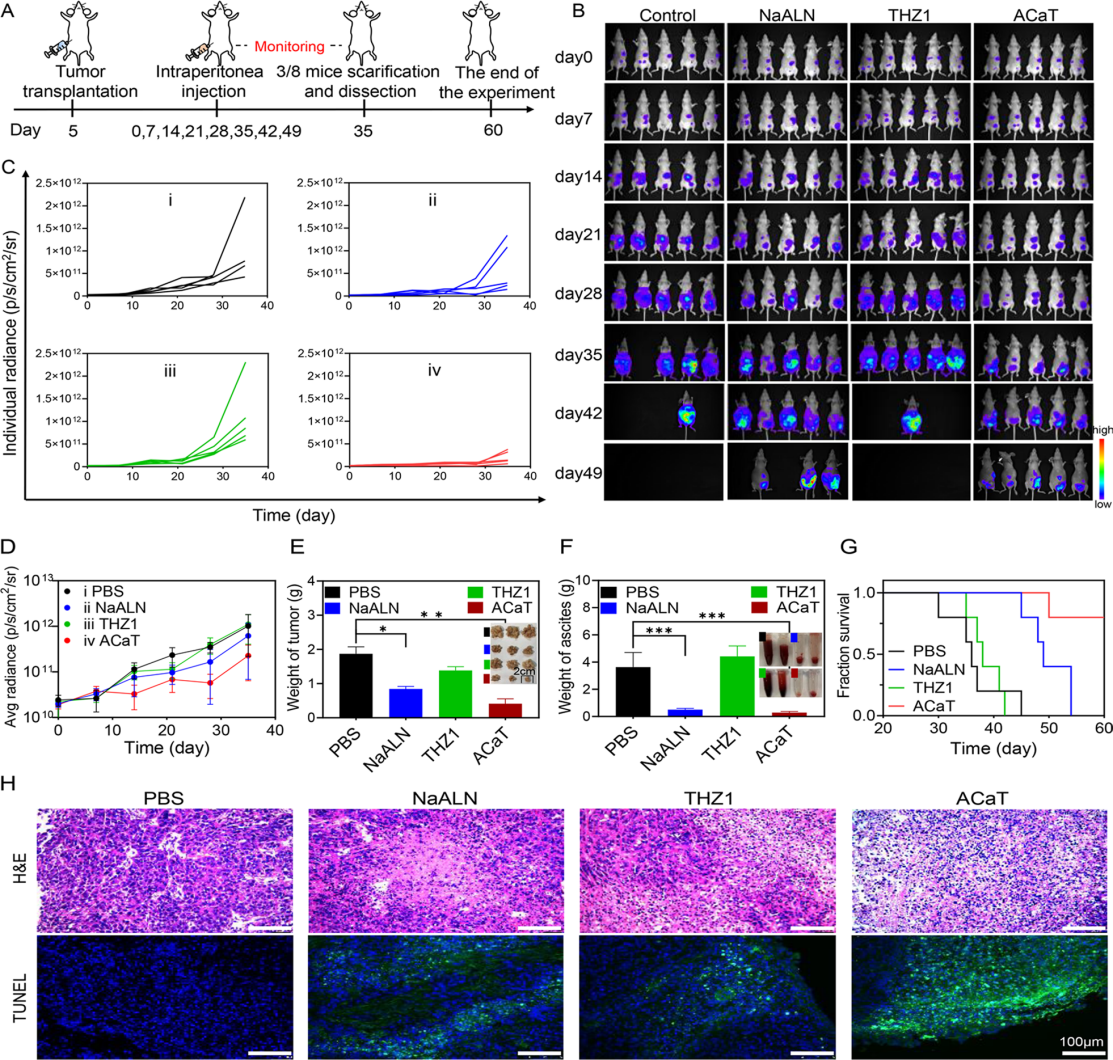
In vivo antitumor effect of ACaT on SKOV3 ovarian tumor-bearing mice. **A** The timeline of animal experiment. **B** In vivo bioluminescent images of mice after different treatments (*n* = 5). **C** Individual fluorescence intensity of mice treated with PBS, NaALN, THZ1 and ACaT calculated from the in vivo bioluminescent images. **D** Average fluorescence intensity of mice treated with PBS, NaALN, THZ1 and ACaT (means ± SD, *n* = 5). **E** Tumor weight and representative images of tumor tissues harvested on day 35 (means ± SD, *n* = 3). **F** Ascites weight and representative images of ascites harvested on day 35 (means ± SD, *n* = 3). **G** Kaplan–Meier survival curves of mice in the different treatment groups. **H** H&E and TUNEL staining images of tumor tissues for each group after 35 days treatment. Scale bar: 100 μm

Encouraged by the strong antitumor activity and tumor cell uptake characters in vitro, the anticancer effect of ACaT nanomedicine in intraperitoneal xenograft mouse model was evaluated. The therapeutic schedule of animal experiment was shown in Fig. 5A. After the successful establishment of abdominal tumor model, the mice were randomly divided into four groups with eight mice in each group. Treatment was performed every week for a total of 8 weeks. On day 35, three mice in each group were killed, and the tumor weight and ascites weight were recorded. The experiment was ended on day 60 and all mice were euthanized. According to the quantitative results of fluorescence intensity, ACaT group had the best inhibitory effect on tumor growth, while NaALN had a modest inhibitory effect on tumor growth compared with the control group (Fig. 5B, C). Nevertheless, the bioluminescent value of THZ1 group was similar to that of the control group, implying that THZ1 could not inhibit tumor growth (Fig. 5B–D). It was probably because THZ1 had low bioavailability and short half-life in vivo.

At 35 days post administration, ACaT group exhibited a strong antitumor effect by inhibiting tumor growth and ascites formation (Fig. 5E, F). It could also be seen from abdominal circumference data (Additional file 1: Fig. S15) wherein abdominal circumference of the mice in PBS and THZ1 groups increased significantly at the later stage, which was consistent with the volumes of ascites. The average survival time of mice was 36 days in PBS group but prominently prolonged in ACaT treatment group (Fig. 5G). 4/5 mice survived at the end of the experiment. H&E and TUNEL staining were used to further confirm the cell necrosis and apoptosis in tumor tissue of ACaT group (Fig. 5H). These results demonstrated that ACaT nanomedicine showed higher antitumor activity than THZ1 and NaALN in an intraperitoneal xenograft model with human ovarian cancer. All treated mice behaved normally, and their weights were well maintained, suggesting good biocompatibility of ACaT (Additional file 1: Fig. S16).

### Biocompatibility of ACaT nanomedicine

**Fig. 6:**
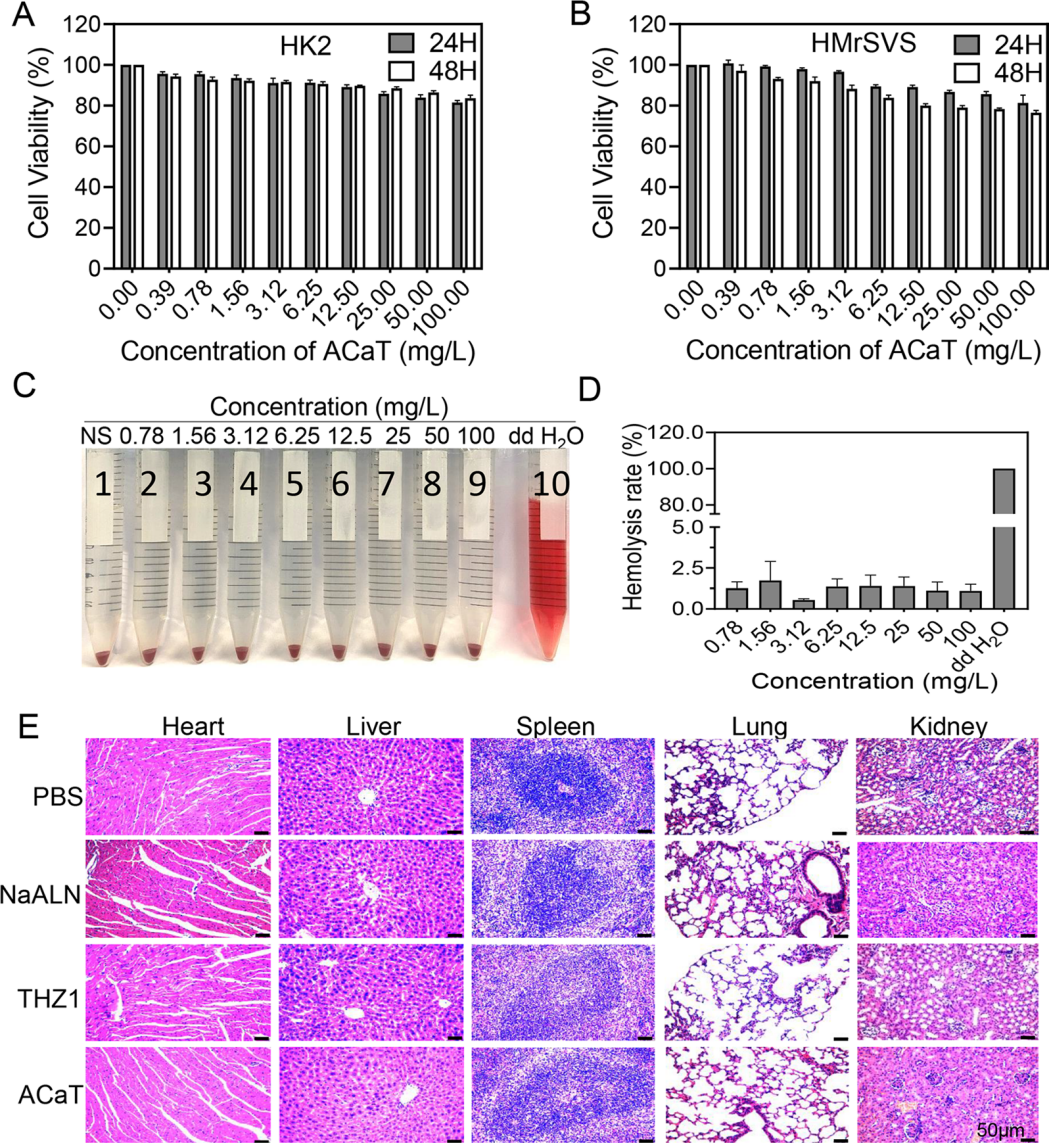
The biosafety evaluation of ACaT in vitro and in vivo. Cell viability of **A** HK2 cells and **B** HMrSVS cells cultured with various concentrations of ACaT for 24 and 48 h, respectively. **C** The photographs of human erythrocytes treated with ACaT at different concentrations. Samples 1–10: negative control (NS), eight different concentrations of ACaT, positive control (dd H_2_O). **D** Hemolysis rate of ACaT, NS and dd H_2_O. **E** H&E staining of tissue slice of main organs in PBS, NaALN, THZ1 and ACaT groups (*n* = 3). Scale bar, 50 μm

The biocompatibility of ACaT was systematically evaluated. The toxicities of ACaT on HK2 and HMrSVS were determined by CCK8 assay. As shown in Fig. 6A, B, ACaT had no significant cytotoxic effect on human normal cells HK2 and HMrSVS. In vitro hemolysis test was a general method to evaluate the blood compatibility of nanomedicine. Hemolysis is defined as the hemolysis rate of human erythrocytes exceeding 5%. As shown in Fig. 6C, D, the hemolysis rates of each ACaT concentration were less than 5%, and no signs of hemolysis were observed even at the high concentration (100 mg/ L). Finally, the histocompatibility of ACaT were evaluated by H&E staining. After 8 cycles of treatments, the tumor bearing mice were sacrificed and the major organs were extracted. As can be seen from Fig. 6E, the heart, liver, spleen, lung and kidney of mice in NaALN, THZ1 and ACaT groups had no obvious histological damage, indicating the excellent biocompatibility of as-synthesized ACaT nanomedicine both in vitro and in vivo.

## Conclusions

In summary, a novel ACaT nanomedicine was designed and successfully synthesized by self-assembly of alendronate, calcium and small molecule inhibitor THZ1 via coordination polymerization and hydrophobic forces. It had high drug loading rate and good stability in physiological environment. After being internalized by tumor cells, ACaT induced cell cycle arrest, apoptosis and inhibited cell migration by releasing anticancer drugs. Its antitumor properties and biocompatibility had been systematically evaluated by in vitro screening assays. In an intraperitoneal xenograft model with human ovarian cancer, intraperitoneal ACaT could accurately target tumors, effectively suppress tumor growth, and significantly inhibit ascites production. In summary, our work provides a versatile carrier-free nanomedicine platform for the treatment of advanced ovarian cancer.

## Supplementary Information

**Additional file 1: Figure S1.** XPS spectra of the ACaT samples. **Figure S2.** In vitro THZ1 release curves in PBS with different pH values. **Figure S3.** Transcriptome sequencing and bioinformatics analyses for ovarian cancer cells SKOV3 treated with PBS (control), THZ1 (0.5 μM), NaALN (850 μM) and ACaT (100 mg/L) for 24 h. **Figure S4.** KEGG pathway analysis of downregulated genes in SKOV3 cells treated with ACaT (100 mg/L) for 24 h. **Figure S5.** KEGG Focal Adhesion signaling pathway (04510) of SKOV3 cells treated with ACaT (100 mg/L) for 24 h. **Figure S6.**  Cell cycle analysis of SKOV3 cells treated with PBS (control), NaALN (400 μM), THZ1 (0.25 μM), ACaT (12.5 mg/L) for 24 h by flow cytometry. **Figure S7.** ROS generation analysis of SKOV3 cells treated with THZ1 at concentrations of 0, 0.25, 0.5 and 1 μM for 24 h by flow cytometry. **Figure S8.** The intracellular calcium ions assay of SKOV3 cells treated with PBS (control),  NaALN (200 μM), THZ1 (0.01 μM) and ACaT (1.56 mg/L) for 48 h. Scale bar, 100 μm. **Figure S9. **The mitochondrial membrane potential analyses of  SKOV3 cells treated with PBS (control), NaALN (850 μM), THZ1 (0.5 μM) and ACaT (100 mg/L) for 6 h by flow cytometry using JC-1 staining. **Figure S10.** Cell viability analysis of SKOV3 cells treated with CaCl_2_ at different concentrations for 24 and 48 h. **Figure S11.** Quantitative data from scratch assay of SKOV3 cells treated with PBS (control), NaALN (200 μM), THZ1 (0.01 μM) and ACaT (6.25 mg/L) for 24 h. The wound gap% were plotted by *GraphPad* Prism 8.0. **Figure S12.** Construction of intraperitoneally disseminated ovarian tumor xenograft model on mouse. **Figure S13.** Anatomical bioluminescent images and bright fields of liver and spleen metastases (Arrows indicate the tumors). **Figure S14.** Fluorescent photographs of organs and tumors of dissected mice at 72 h post-injection. **Figure S15.** Abdominal girth changes of SKOV3 tumor-bearing mice in different treatment groups (*n* = 5). **Figure S16.** Body weight changes of SKOV3 tumor-bearing mice in different treatment groups (*n* = 5).
