# Supplementary material for: Carrier-free multifunctional nanomedicine for intraperitoneal disseminated ovarian cancer therapy
Source: J Nanobiotechnology. 2022 Feb 22;20:93. doi: 10.1186/s12951-022-01300-4 (PMC8864853; doi:10.1186/s12951-022-01300-4)
Supplement: Supplementary file 1 — Additional file 1: Figure S1. XPS spectra of the ACaT samples. Figure S2. In vitro THZ1 release curves in PBS with different pH values. Figure S3. Transcriptome sequencing and bioinformatics analyses for ovarian cancer cells SKOV3 treated with PBS (control), THZ1 (0.5 μM), NaALN (850 μM) and ACaT (100 mg/L) for 24 h. Figure S4. KEGG pathway analysis of downregulated genes in SKOV3 cells treated with ACaT (100 mg/L) for 24 h. Figure S5. KEGG Focal Adhesion signaling pathway (04510) of SKOV3 cells treated with ACaT (100 mg/L) for 24 h. Figure S6. Cell cycle analysis of SKOV3 cells treated with PBS (control), NaALN (400 μM), THZ1 (0.25 μM), ACaT (12.5 mg/L) for 24 h by flow cytometry. Figure S7. ROS generation analysis of SKOV3 cells treated with THZ1 at concentrations of 0, 0.25, 0.5 and 1 μM for 24 h by flow cytometry. Figure S8. The intracellular calcium ions assay of SKOV3 cells treated with PBS (control), NaALN (200 μM), THZ1 (0.01 μM) and ACaT (1.56 mg/L) for 48 h. Scale bar, 100 μm. Figure S9. The mitochondrial membrane potential analyses of SKOV3 cells treated with PBS (control), NaALN (850 μM), THZ1 (0.5 μM) and ACaT (100 mg/L) for 6 h by flow cytometry using JC-1 staining. Figure S10. Cell viability analysis of SKOV3 cells treated with CaCl2 at different concentrations for 24 and 48 h. Figure S11. Quantitative data from scratch assay of SKOV3 cells treated with PBS (control), NaALN (200 μM), THZ1 (0.01 μM) and ACaT (6.25 mg/L) for 24 h. The wound gap% were plotted by GraphPad Prism 8.0. Figure S12. Construction of intraperitoneally disseminated ovarian tumor xenograft model on mouse. Figure S13. Anatomical bioluminescent images and bright fields of liver and spleen metastases (Arrows indicate the tumors). Figure S14. Fluorescent photographs of organs and tumors of dissected mice at 72 h post-injection. Figure S15. Abdominal girth changes of SKOV3 tumor-bearing mice in different treatment groups (n = 5). Figure S16. Body weight changes of SKOV3 tumor-bearing m [file 12951_2022_1300_MOESM1_ESM.docx]

Supporting Information

Carrier-free multifunctional nanomedicine for intraperitoneal disseminated ovarian cancer therapy

*Xiuyu Huang^1^*^†^*, Miaojuan Qiu^1^*^†^*, Tianqi Wang^2^, Binbin Li^1^, Shiqiang Zhang^1^, Tianzhi Zhang^1^, Peng Liu^1^, Qiang Wang^1^, Zhi Rong Qian^1^, Chengming Zhu^1^*, Meiying Wu^2^* and Jing Zhao^1^**

*^1^* The Seventh Affiliated Hospital of Sun Yat-sen University, Sun Yat-sen University, Shenzhen, 518107, Guangdong, P. R. China.

*^2^* School of Pharmaceutical Sciences (Shenzhen), Sun Yat-sen University, Shenzhen, 518107, Guangdong, P. R. China.

E-mail addresses:

zhuchm3@mail.sysu.edu.cn (C. Zhu)

wumy53@mail.sysu.edu.cn (M. Wu)

zhaoj265@mail.sysu.edu.cn (J. Zhao)

^†^ These authors contributed equally to this work.


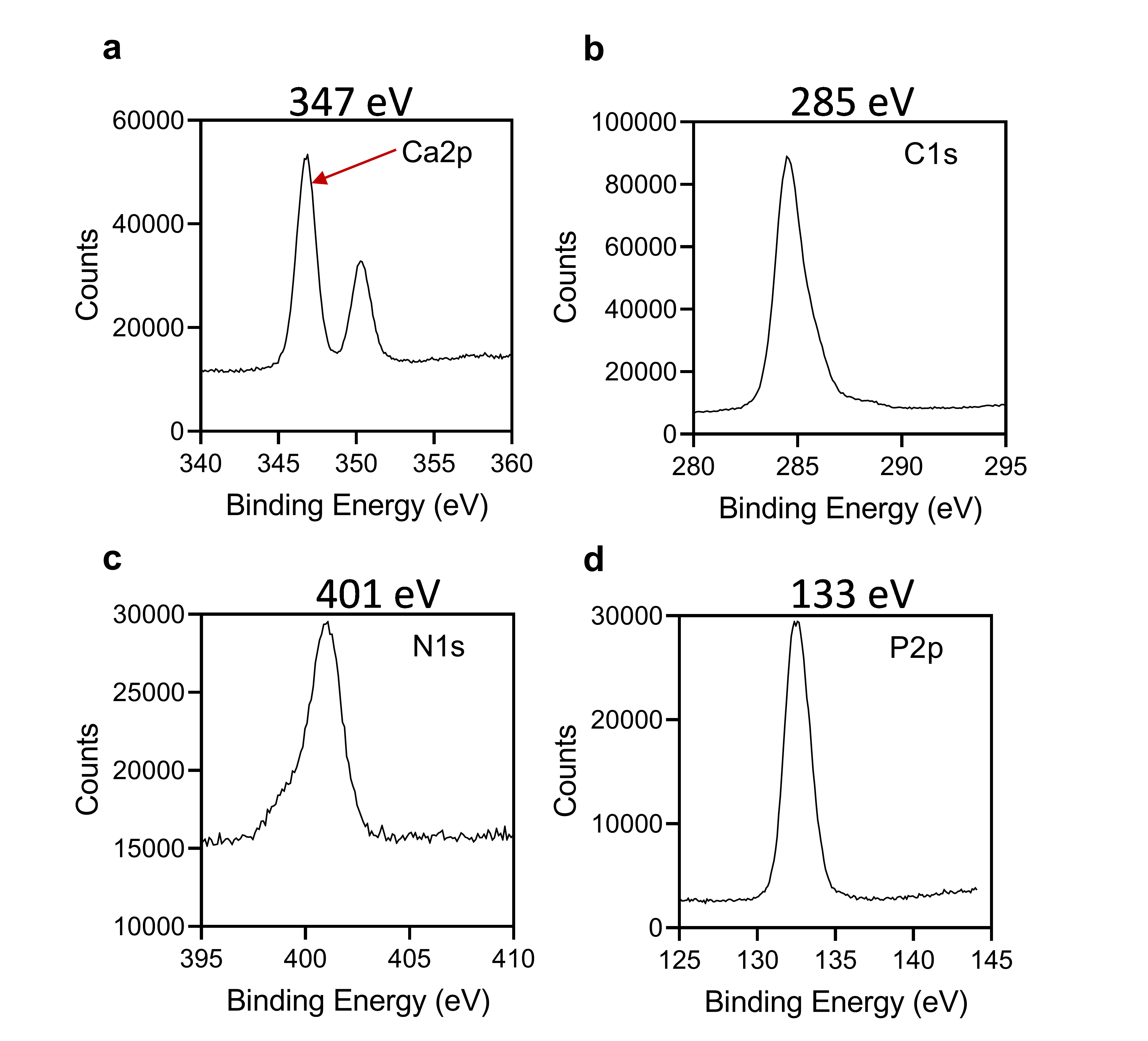


**Additional file 1: Figure S1.** XPS spectra of the ACaT samples. (a) Ca2p XPS spectra; (b) C1s XPS spectra; (c) N1s XPS spectra; (d) P2p XPS spectra.

**
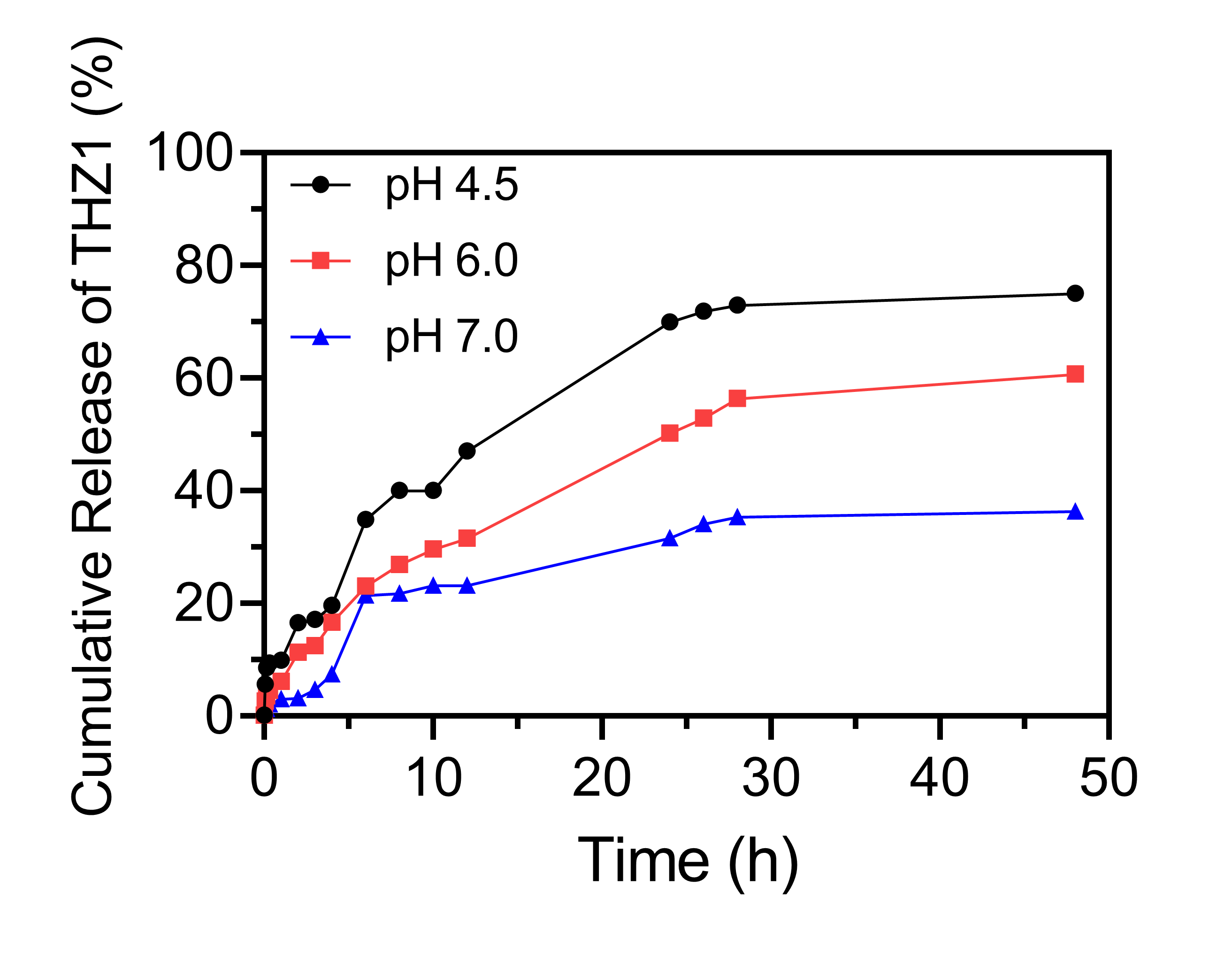
**

**Additional file 1: Figure S2.** *In vitro* THZ1 release curves in PBS with different pH values. 10 mg ACaT was dispersed in 10 mL PBS (pH = 4.5, 6.0, 7.4) at 37°C. Culture on a shaker for 48 h at a constant speed. 100 μL of release media was extracted at different time points (0 min, 5 min, 10 min, 20 min, 30 min, 60 min, 1 h, 2 h, 3 h, 4 h, 6 h, 8 h, 10 h,12 h, 24 h, 26 h, 28 h and 48 h) Supernatant was removed after centrifugation. The amount of drug release from THZ1 was measured by HPLC (Shimadzu LC20AB, Japan). Experimental conditions: C18 column (100 mm × 4.6 mm, particle size, 5 μm); Column temperature: 25 °C; Flow rate: 1.0 mL/min; Detection: 254 nm; Injection volume: 20 μL. HPLC conditions are as follows: eluent A (water) and eluent B (MeOH, v/v).


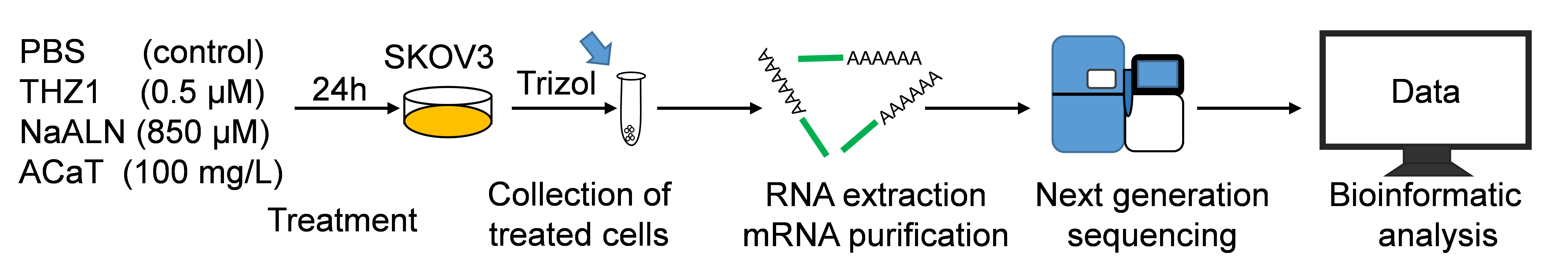


**Additional file 1: Figure S3.** Transcriptome sequencing and bioinformatics analyses for ovarian cancer cells SKOV3 treated with PBS (control), THZ1 (0.5 μM), NaALN (850 μM) and ACaT (100 mg/L) for 24 h. Then cells were washed three times with ice-cold PBS and lysed by the Trizol reagent at a ratio 10 cm^2^/mL with incubation of 5 min at room temperature. RNA was purified by RNA purification kit (Thermo Fisher Scientific). RNA-seq libraries were generated and sequenced following the standard mRNA protocols on a Hiseq 2500 (Illumina, PE150). Clean reads were obtained from the raw reads by removing the adaptor sequences, low quality sequences and reads containing poly-N with Trimmomatic software.

**
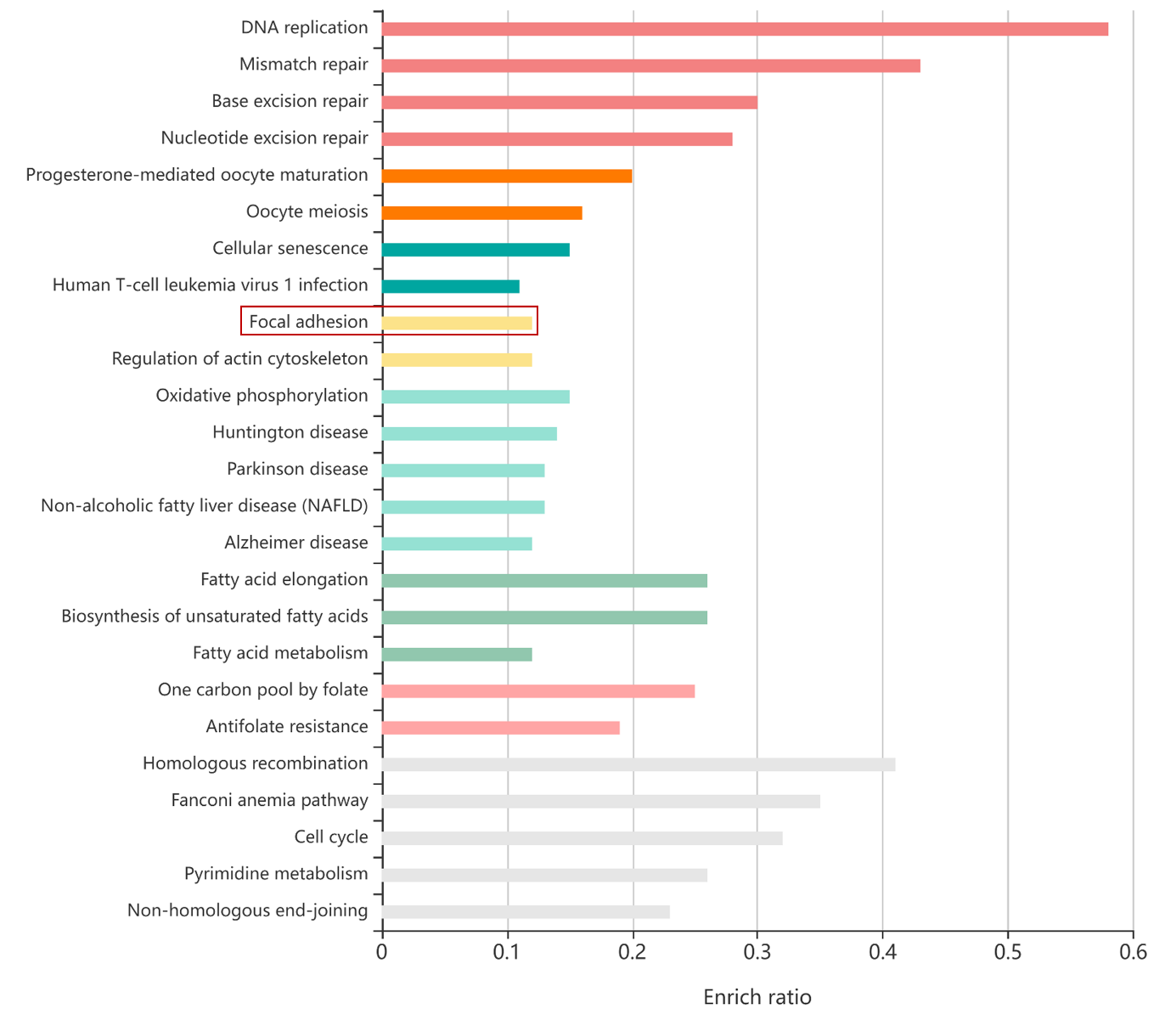
**

**Additional file 1: Figure S4.** KEGG pathway analysis of downregulated genes in SKOV3 cells treated with ACaT (100 mg/L) for 24 h. Each row represents an enriched function, and the length of the bar represents the enrich ratio, which is calculated as "downregulated gene number"/ "backgound gene number". Different colors represent clusters with different functions. Inside the red box is pathway associated with migration.

**
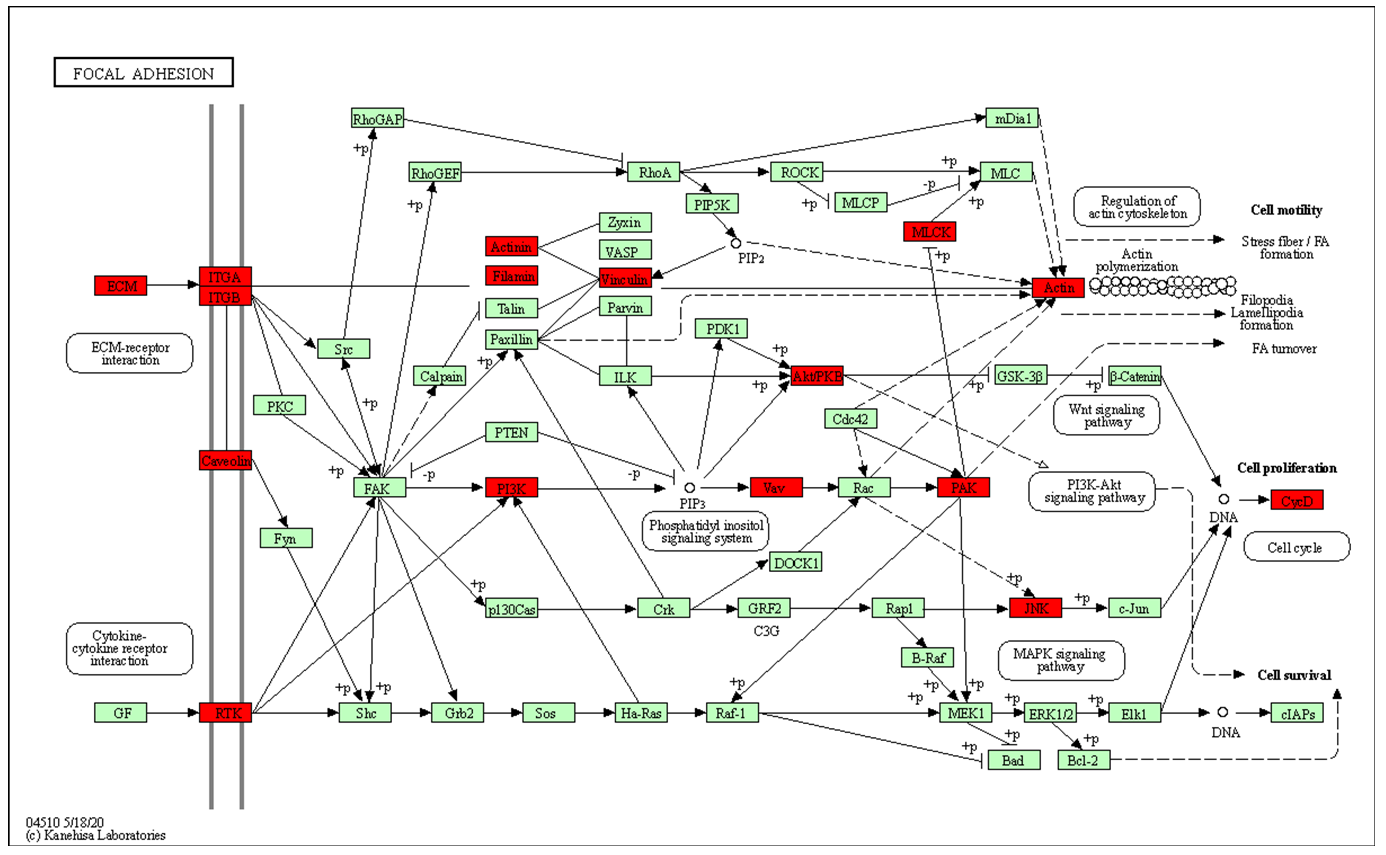
**

**Additional file 1: Figure S5.** KEGG Focal Adhesion signaling pathway (04510) of SKOV3 cells treated with ACaT (100 mg/L) for 24 h. Data were retrieved from the KEGG database (<https://www.genome.jp/kegg/>). Red boxes represent downregulated genes and green boxes indicate background genes.

**
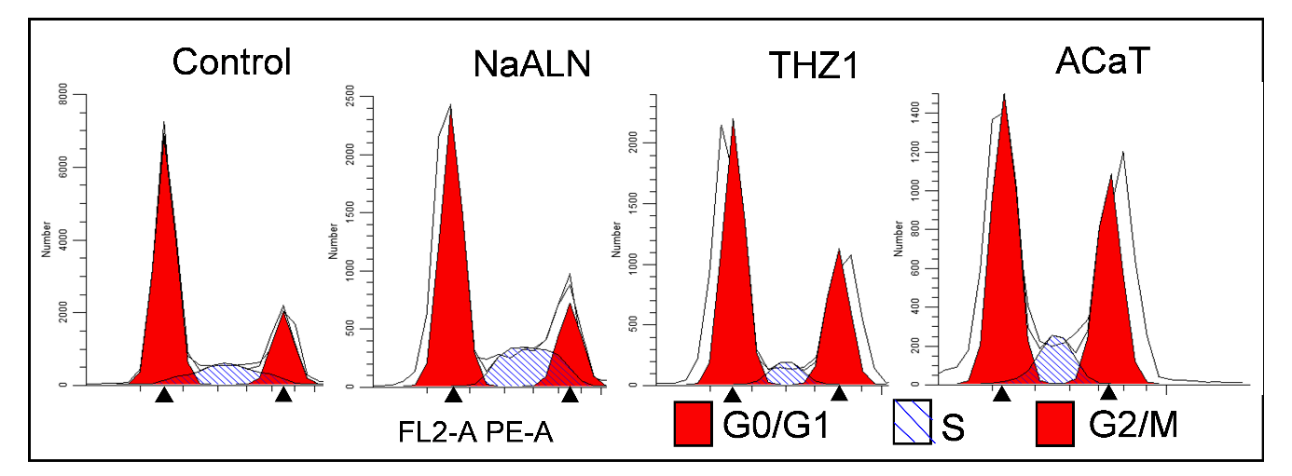
**

**Additional file 1: Figure S6.** Cell cycle analysis of SKOV3 cells treated with PBS (control), NaALN (400 μM), THZ1 (0.25 μM), ACaT (12.5 mg/L) for 24 h by flow cytometry. Percentages of G0/G1, S and G2/M phase cells were determined using ModFit LT5.0 software.

**
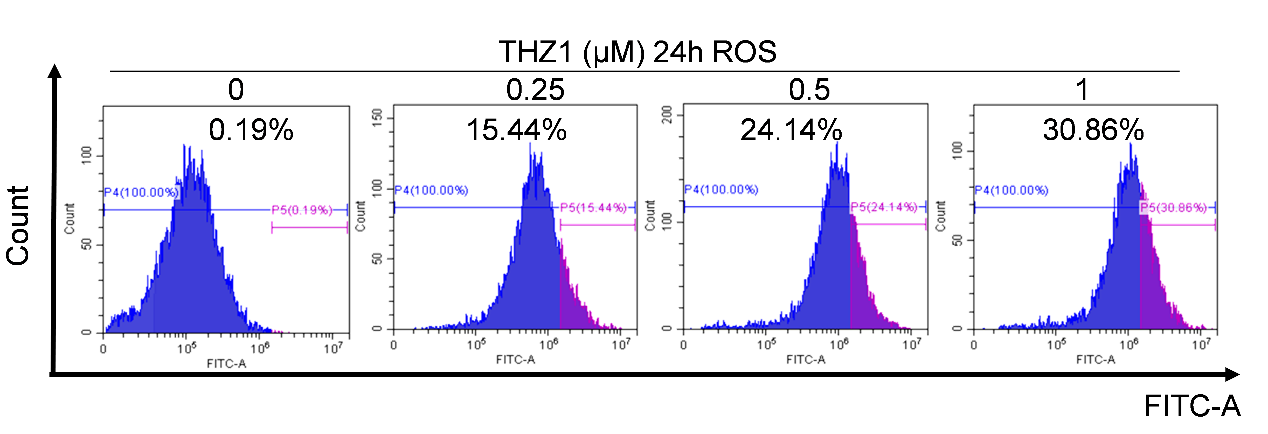
**

**Additional file 1: Figure S7.** ROS generation analysis of SKOV3 cells treated with THZ1 at concentrations of 0, 0.25, 0.5 and 1 μM for 24 h by flow cytometry.

**
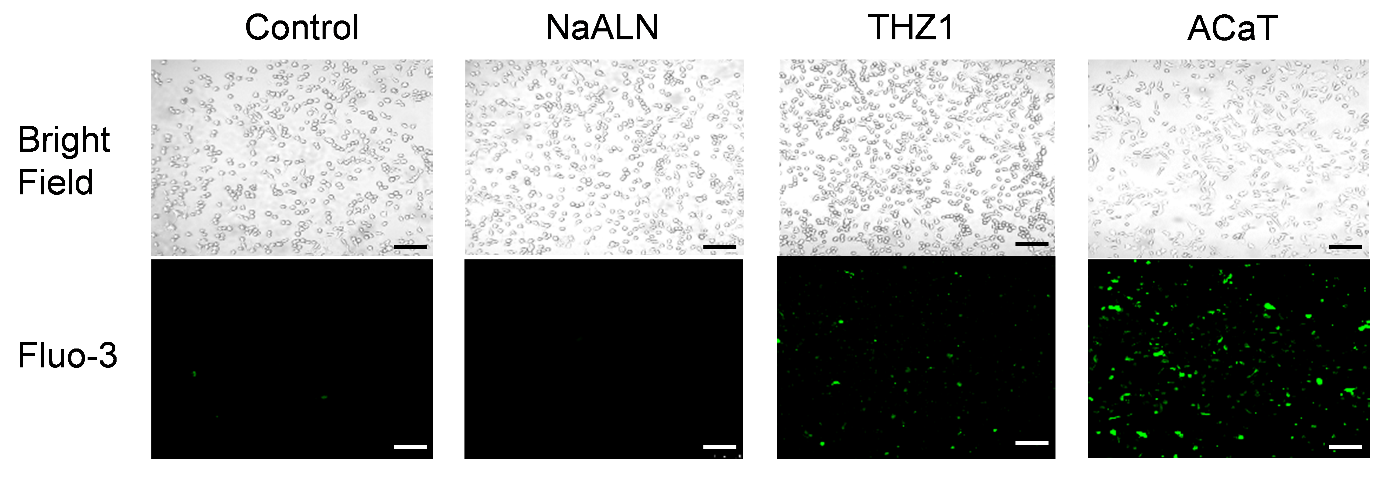
**

**Additional file 1: Figure S8.** The intracellular calcium ions assay of SKOV3 cells treated with PBS (control),  NaALN (200 μM), THZ1(0.01 μM) and ACaT (1.56 mg/L) for 48 h. Scale bar, 100 μm.

**
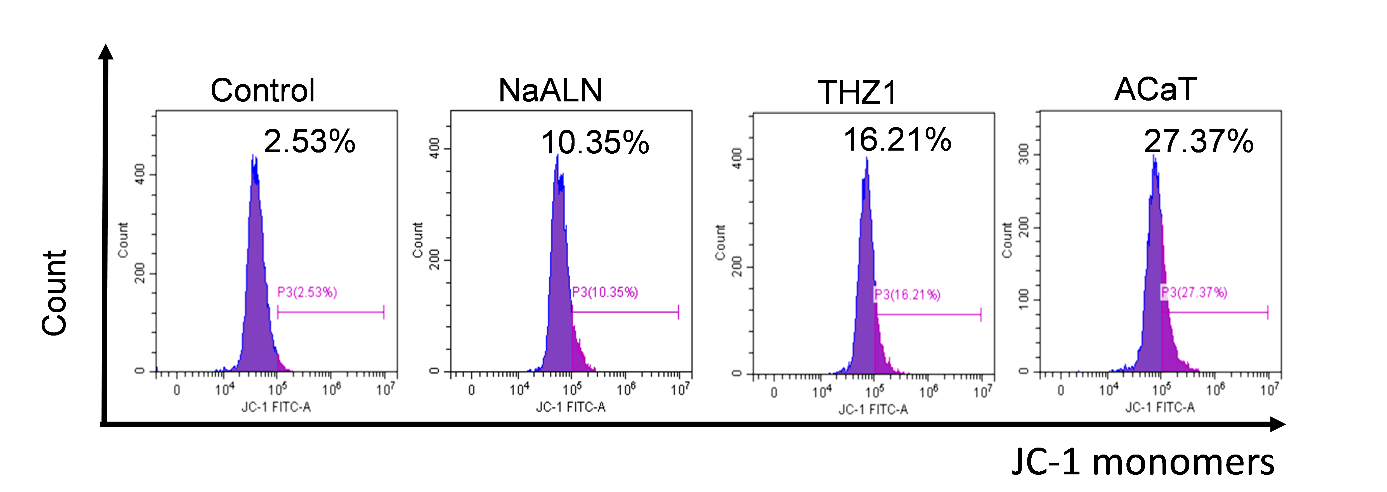
**

**Additional file 1: Figure S9.** The mitochondrial membrane potential analyses of  SKOV3 cells treated with PBS (control), NaALN (850 μM), THZ1 (0.5 μM) and ACaT (100 mg/L) for 6 h by flow cytometry using JC-1 staining.

**
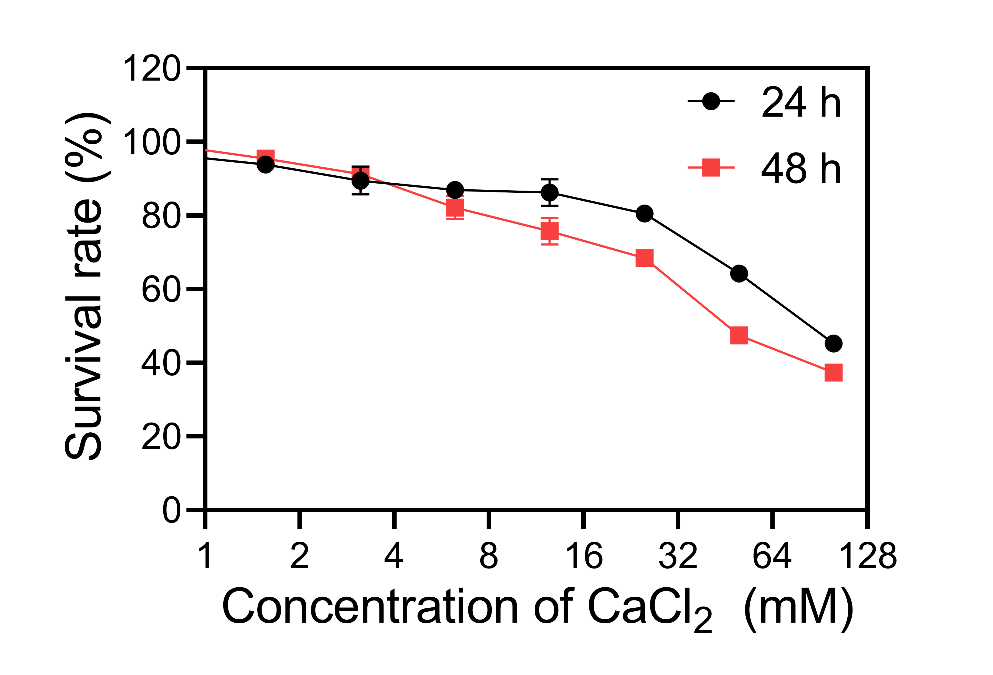
**

**Additional file 1: Figure S10.** Cell viability analysis of SKOV3 cells treated with CaCl_2_ at different concentrations for 24 and 48 h. The cell survival curves were plotted by *GraphPad* Prism 8.0.

**
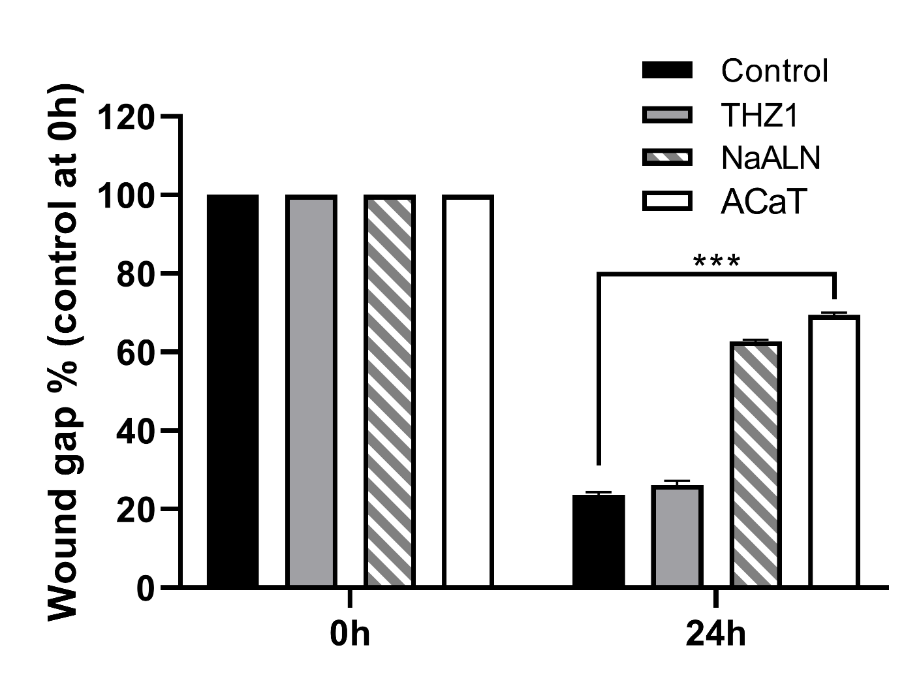
**

**Additional file 1: Figure S11.** Quantitative data from scratch assay of SKOV3 cells treated with PBS (control), NaALN (200 μM), THZ1 (0.01 μM) and ACaT (6.25 mg/L) for 24 h. The wound gap% were plotted by *GraphPad* Prism 8.0.

**
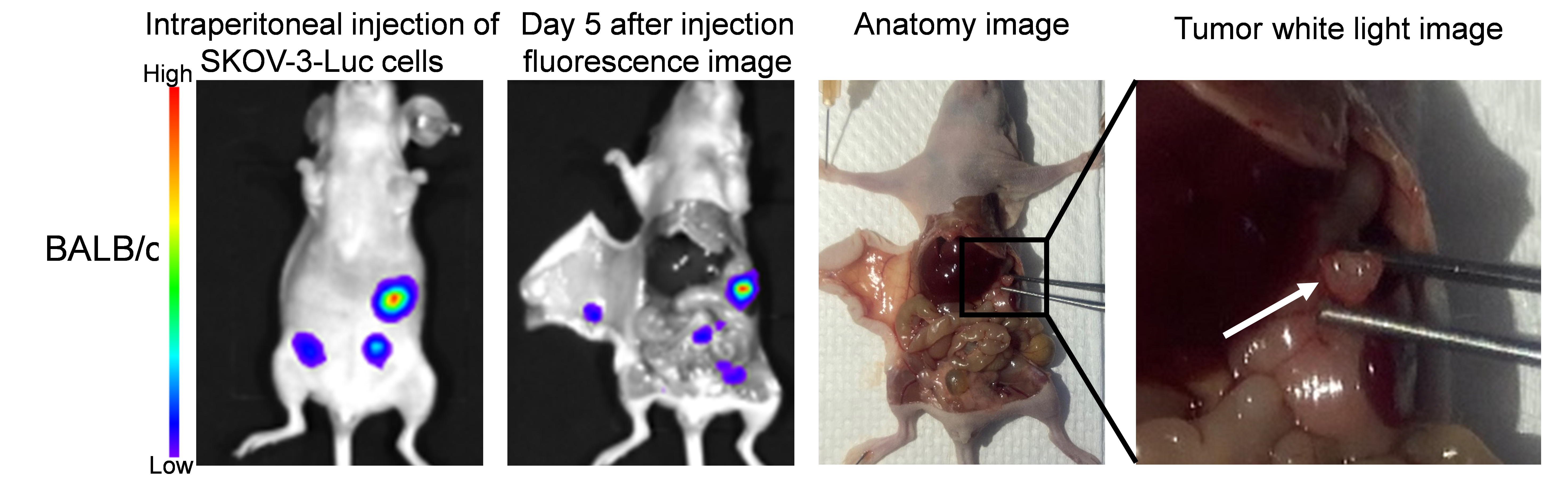
**

**Additional file 1: Figure S12.** Construction of intraperitoneally disseminated ovarian tumor xenograft model on mouse. SKOV3 cells (5 🞨 10^6^ cells/400 μL in PBS) were injected intraperitoneally into BALB/c nude mouse. After 5 day, the mice were killed and dissected for observing tumor location and size.

**
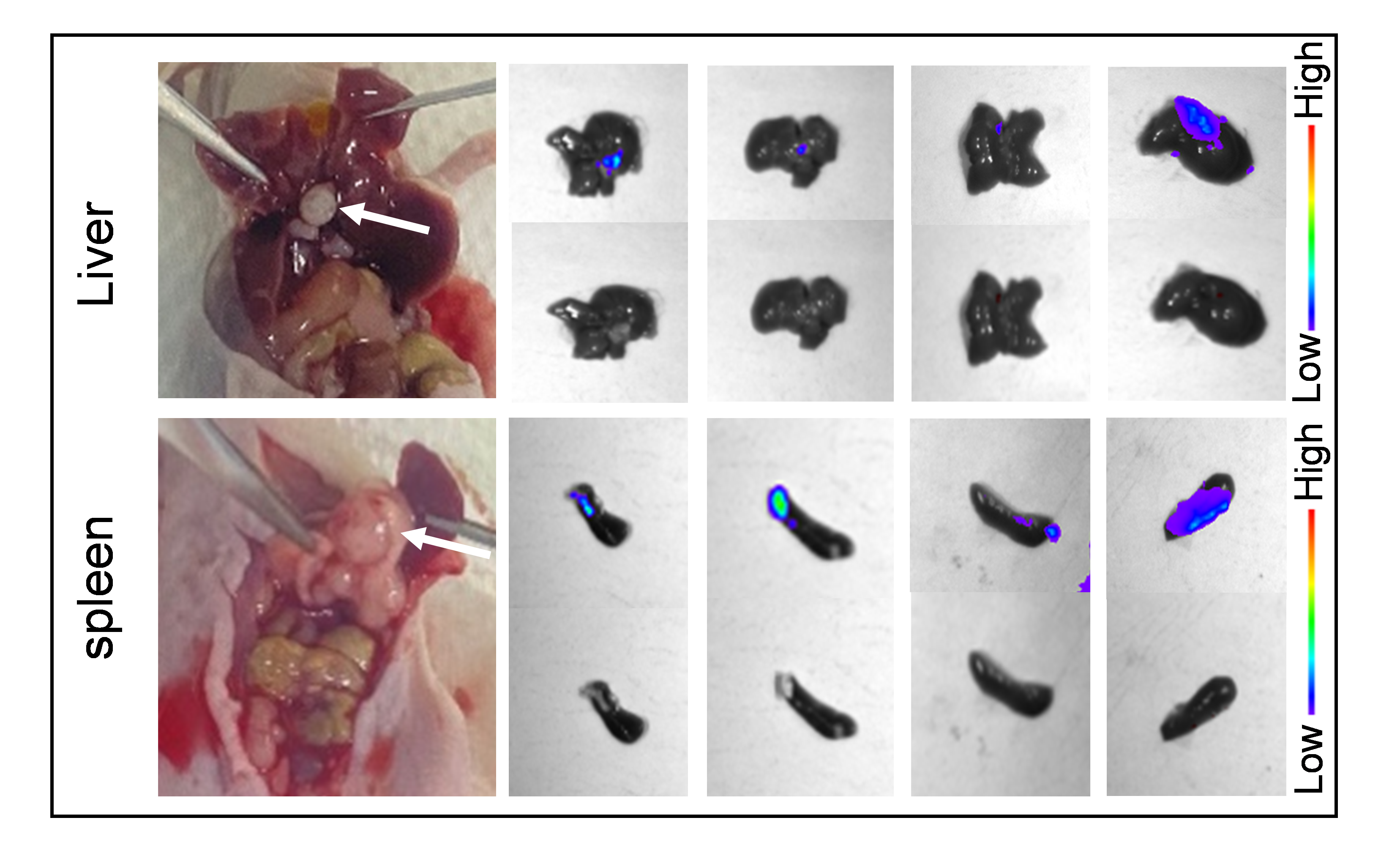
**

**Additional file 1: Figure S13.** Anatomical bioluminescence images and bright fields of liver and spleen metastases (Arrows indicate the tumors). SKOV3-*Luc* cells (5 🞨 10^6^ cells/400 μL in PBS) were injected intraperitoneally into BALB/c nude mouse. After 30 day, the mice were killed and dissected for observing tumor location and size. 4 of 10 mice treated with PBS had liver and spleen metastases. 1 of 10 had both liver and spleen metastases.

**
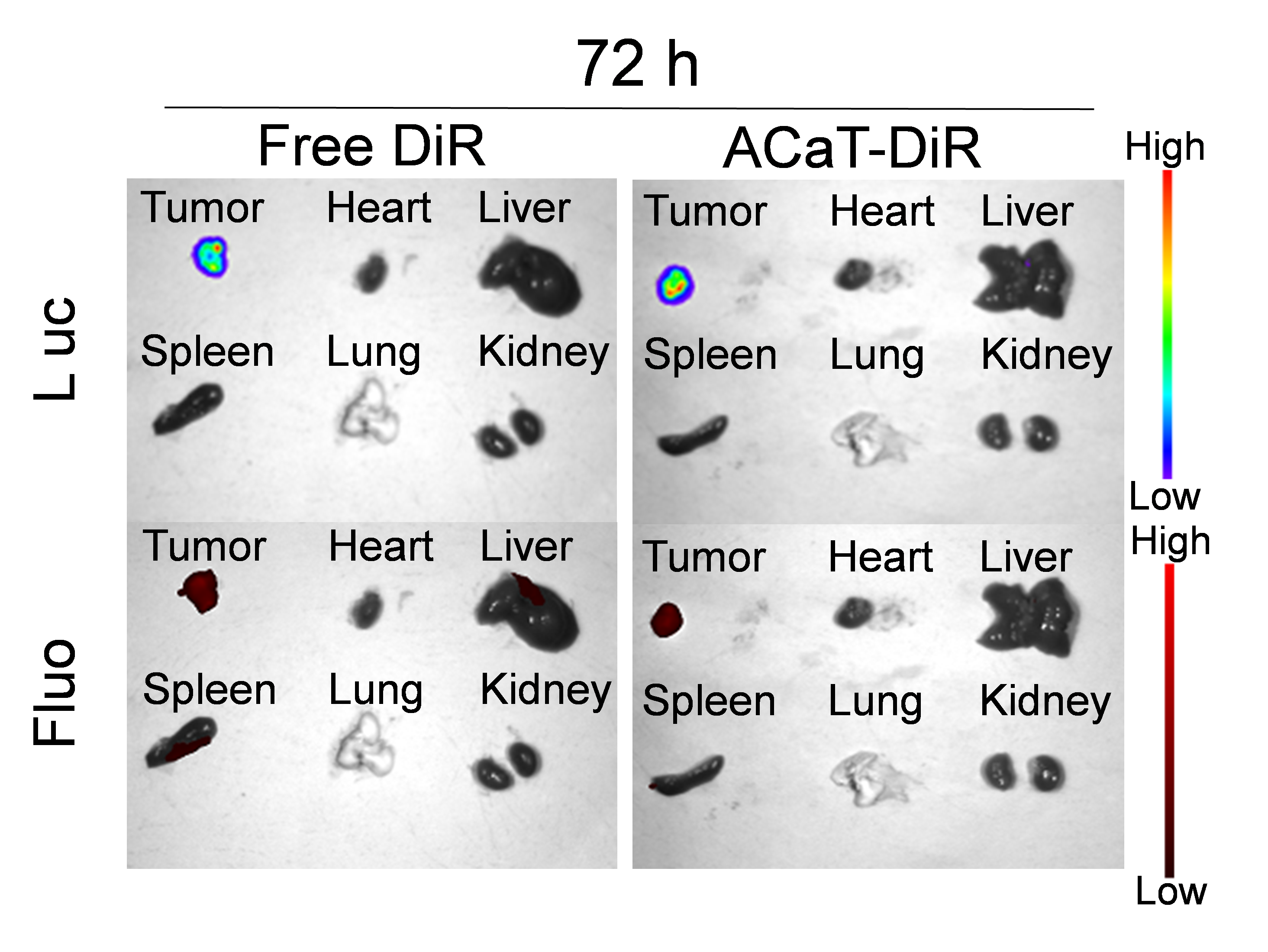
**

**Additional file 1: Figure S14.** Fluorescent photographs of organs and tumors of dissected mice at 72 h post-injection. The images on the top were tumor bioluminescence and bottom were DiR dye fluorescence.

**
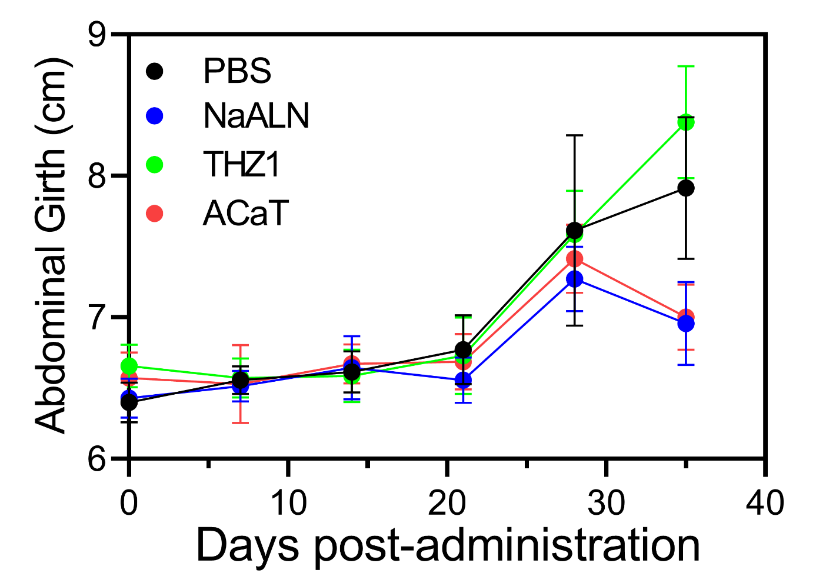
**

**Additional file 1: Figure S15.** Abdominal girth changes of SKOV3 tumor-bearing mice in different treatment groups *(n = 5)*.

**
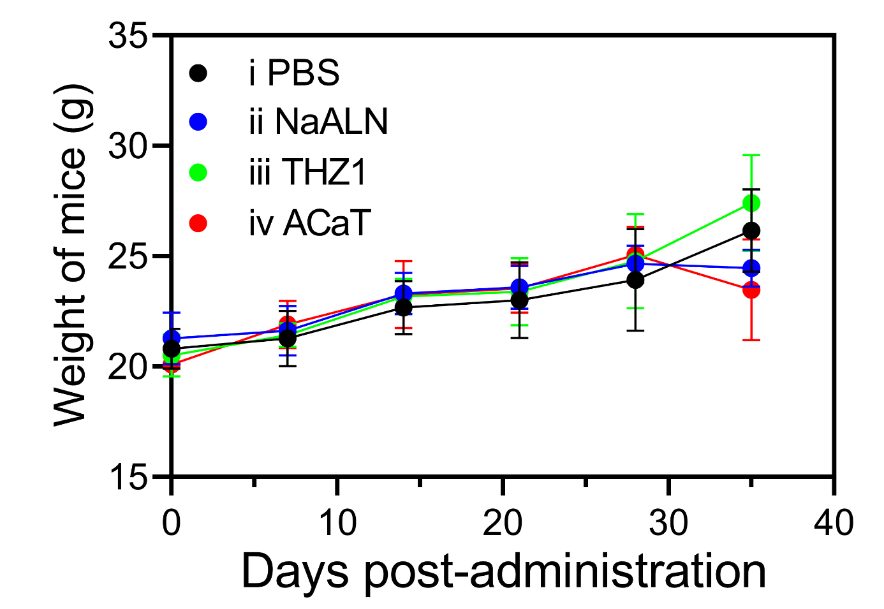
**

**Additional file 1: Figure S16.** Body weight changes of SKOV3 tumor-bearing mice in different treatment groups (*n* = 5) .
